# Socio-ecological costs of Amazon nut and timber production at community household forests in the Bolivian Amazon

**DOI:** 10.1371/journal.pone.0170594

**Published:** 2017-02-24

**Authors:** Marlene Soriano, Frits Mohren, Nataly Ascarrunz, Wolfram Dressler, Marielos Peña-Claros

**Affiliations:** 1 Instituto Boliviano de Investigación Forestal, Santa Cruz, Bolivia; 2 Forest Ecology and Forest Management Group, Wageningen University, Wageningen, The Netherlands; 3 School of Geography, University of Melbourne, Melbourne, Australia; New York State Museum, UNITED STATES

## Abstract

The Bolivian Amazon holds a complex configuration of people and forested landscapes in which communities hold secure tenure rights over a rich ecosystem offering a range of livelihood income opportunities. A large share of this income is derived from Amazon nut (*Bertholletia excelsa*). Many communities also have long-standing experience with community timber management plans. However, livelihood needs and desires for better living conditions may continue to place these resources under considerable stress as income needs and opportunities intensify and diversify. We aim to identify the socioeconomic and biophysical factors determining the income from forests, husbandry, off-farm and two keystone forest products (i.e., Amazon nut and timber) in the Bolivian Amazon region. We used structural equation modelling tools to account for the complex inter-relationships between socioeconomic and biophysical factors in predicting each source of income. The potential exists to increase incomes from existing livelihood activities in ways that reduce dependency upon forest resources. For example, changes in off-farm income sources can act to increase or decrease forest incomes. Market accessibility, social, financial, and natural and physical assets determined the amount of income community households could derive from Amazon nut and timber. Factors related to community households’ local ecological knowledge, such as the number of non-timber forest products harvested and the number of management practices applied to enhance Amazon nut production, defined the amount of income these households could derive from Amazon nut and timber, respectively. The (inter) relationships found among socioeconomic and biophysical factors over income shed light on ways to improve forest-dependent livelihoods in the Bolivian Amazon. We believe that our analysis could be applicable to other contexts throughout the tropics as well.

## Introduction

The contribution of forests to rural livelihoods is well-acknowledged throughout the tropics [1–6]. In particular, the local provision of, and the financial benefits from, timber and non-timber forest products (NTFPs) play an important role in improving rural livelihoods while also preventing forest degradation and deforestation [7]. Yet, high dependency on forest income can potentially ‘trap’ rural families in cycles of poverty due to low prices caused by insecure forest tenure and poor access to markets [8,9]. Under improved socioeconomic and biophysical conditions, however, a greater value can be drawn from forest resources with the potential to increase the income and living conditions of rural families [1,2]. Recent studies show that income from the forest increases when rural families harvest a larger set of forest products [2], and have improved organization [10] and road infrastructure [1]. The influence of these and other socioeconomic and biophysical factors on rural livelihoods have been examined in the context of changing rural economies. More specifically, we investigated how such factors are shaping the various sources of income derived by community households in the Bolivian Amazon, focusing on two keystone forest products: Amazon nut (a.k.a. Brazil nut, *Bertholletia excelsa*) and timber. We address these questions by combining socioeconomic information of community households and ecological information of household forests.

Changes in the demography of harvested species in response to socioeconomic factors have been examined by combining structured interviews at the household level with biological inventories at the community-level (S1 Table). Uma Shaanker et al. [11] pioneered this approach by differentiating three main socioeconomic attributes: i) extent of dependence, ii) local ecological knowledge and iii) market organization. Each attribute encompasses several socioeconomic variables for calculating the ecological costs of NTFP use in India. Contemporary researchers such as Brown et al. [12], Mutenje et al. [13] and Steele et al. [14] adopted Uma Shaanker et al.’s approach to look at the ecological costs of firewood use in African countries (S1 Table). Their findings offer new insights to the understanding of the patterns of resource use and of changes in the availability of forest resources. In the Neotropics, only Zeidemann et al. [15] have examined the socioeconomic factors governing Amazon nut harvesting intensity and found that access to the market increased fruit production of individual trees and the income derived from Amazon nut. Up to now, few studies have examined the potential socio-ecological costs of harvesting multiple forest products in relation to their impact on rural livelihoods. Furthermore, none of these studies addressed this topic at the household and household forest levels [16]. In the Bolivian Amazon region, we found an area that offers a unique opportunity to fulfill this knowledge gap.

The communities and households in the Bolivian Amazon are becoming more market-oriented due to the increasing accessibility of markets [17] and demands from growing human populations in recent years [18]. The rise in market exchange and need for cash in communities themselves may also modify the use of available forest resources. For example, in 2009, these communities sold 71% more Amazon nut than in 1997 (2821 vs 4811 boxes, i.e., a box containing 23 kg of unshelled nuts) [18], an increase that may have been driven by increased international prices [3] and may have resulted from a higher harvesting intensity. However, concurring with demographic population studies, current harvesting levels of Amazon nut do not represent a threat to the long-term sustainability of this species [19,20] (cf. [21,22]). Indeed, human intervention, such as shifting cultivation [23,24] (hereafter referred as agriculture), large disturbances created by logging (e.g. logging gaps and log landings [25]) and historic cultural practices (e.g. enrichment planting by past human inhabitants) [21,26] may have a positive effect on Amazon nut populations due to the high light requirements this species needs for its regeneration [19,25]. The Amazon nut tree coexists with a large number of timber species, which has led community households to increasingly draw income from timber as well. A comprehensive study carried out at the national level on the forest response to selective logging [27] projected a reduction of timber production in subsequent cutting cycles, implying that current rates of timber harvesting are ecologically and economically unsustainable without the implementation of silviculture. These projections are of great concern, especially in view of the land redistribution process that occurred over the last couple of decades in the region; which have added pressure over these forests.

**Fig 1:**
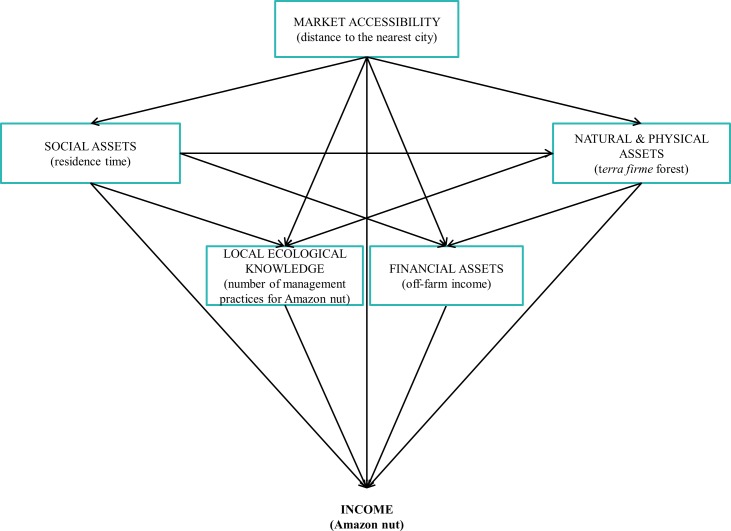
Conceptual framework showing the potential relationships of socioeconomic (i.e., social assets, local ecological knowledge, financial assets) and biophysical attributes (i.e., market accessibility, natural and physical assets) in relation to household income. **Attributes can have direct and indirect effects on the response variable.** An example of these relationships is included within parentheses in each attribute box. This conceptual framework is further developed for timber and Amazon nut income in the Supporting Information. Other variables used to characterize the different attributes are listed in Table 2.

In this study, we aim to identify the socioeconomic and biophysical factors determining the income from forests (timber, NTFPs and hunting), husbandry [agriculture, agroforestry and livestock (mainly chicken and pigs)], off-farm (business, services and gifts) and two keystone forest products (Amazon nut and timber) derived by community households in the Bolivian Amazon region. To this end, we ask three questions. First, what is the contribution of forest to the total income of community households? We expect that the contribution of forest to the total income of community households will be greater than other sources of income (i.e., husbandry and off-farm) due to their high economic dependency on Amazon nut [3,4]. Second, we asked how do socioeconomic and biophysical factors determine forest, husbandry and off-farm incomes derived by community households? We developed a conceptual framework (Fig 1) upon which we hypothesized the following relationships. We expect that mainly asset-based attributes will drive these incomes [28] (e.g., natural and physical assets, see methods for more details). For example, the proportion of *terra firme* or upland forest–highly correlated with land [29,30] and agricultural area [29]–will increase income from forest by comprising more Amazon nut producing trees. Husbandry income on the other hand, will be positively driven by a household’s head residence time [8,29], and negatively driven by the area of *terra firme* forest. Off-farm income will mainly depend on the value of material assets because households in businesses or with a paid job elsewhere will invest in acquiring more assets [29]. Third, we asked more specifically, how do socioeconomic and biophysical factors determine the income that community households draw from Amazon nut and timber? We expect that Amazon nut income will increase with residence time, proportion of working household members [4,11,31], application of a larger number of management practices to increase Amazon nut production, the proportion of *terra firme* forest [3,8]; but will decrease with distance to the nearest city (market) and off-farm income (S1A Fig). Finally, we expect that the income from timber will decrease with distance to the market, and will increase as households carried more specialized tasks within the community timber management plan (CTMP) [3,4], comprised a larger proportion of working members, shared more times their timber benefits, and received greater financial support (S1B Fig). Household forests further away from the market will comprise greater standing timber volume [12].

## Materials and methods

### Research site

The Bolivian Amazon region encompasses the entire Department of Pando and the provinces of Vaca Díez of the Beni Department and Iturralde of the La Paz Department. Approximately 95% of the region is covered by forest [32], and comprises 30% of Bolivia’s timber production forests (8.8 out of 28.8 mill. ha) [33]. Tree diversity ranges from 52–122 species ha^-1^ with a density between 544–627 trees ha^-1^ of trees ≥10 cm diameter at breast height (DBH) [34]. The annual rainfall varies between 1774–1934 mm, while the mean annual temperature differs slightly between the two main regional cities: Cobija (25.4°C) and Riberalta (26.2°C) [35]. The region presents a relatively dry season from May through September with less than 60 mm of precipitation per month. Its topography varies from *terra firme* forests to seasonally flooded areas. *Terra firme* forests comprise over 50% of a forest area [34], and grow on soils with low fertility (i.e., high aluminium toxicity), while seasonally flooded areas have relatively high nutrient-rich soils due to the sediments carried by rivers originating in the Andes [35].

Historically, the economic development of the Bolivian Amazon has depended on NTFP exploitation such as rubber tapping during the late 1890s to early 1980s, and on Amazon nut gathering since the early 1990s [36]. Timber harvesting has increasingly contributed to the regional economy since the 1960s [36]. From around the 1970s, rights to log timber were granted through contracts to timber companies over a pre-determined timber volume [37], and after the enactment of the 1996 Forestry Law, through the granting or concession of a determined area. Contracts and concessions often overlapped with forests that were customarily used for NTFP extraction by rural settlements and indigenous communities [38] who only attained tenure rights in 2008 [39].

The adoption of community timber management plans for timber production (i.e., largely supported by local NGOs and governmental institutions) became an effective way to secure collective tenure rights by already established settlers and temporary Amazon nut gatherers. Despite secure tenure rights, most communities have not fully embraced the legal forestry framework for logging their timber through timber management plans. The implementation of these management plans challenged the capabilities of the newly formed communities in many ways [40], ranging from lack of managerial skills to investments needed for defining the required management [41]. Furthermore, the lack of organizational and negotiation skills have constrained communities from maximizing their benefits from timber and NTFPs [10,42]. Numerous subsequent amendments have been made to the forest management regulations in order to reduce communities’ dependence on external agents and enhance the profits derived from the forest [43]. These modifications created numerous pathways for small-scale timber operations to take place, the most dominant being logging for own use (1996 onwards) and logging of small volumes (2012 onwards). Amendments were also made for NTFPs management, including Amazon nut. As a consequence of these regional dynamics, national policies are currently directed towards integrated land and forest management, which urges forest owners to diversify their forest production to reduce pressure over forest products, but mainly over timber. The context of these management institutions potentially influences harvester decision-making and livelihood outcomes at the household level.

Bolivia’s Amazonian forests have faced dramatic changes since the late 1990s after the implementation of the Forestry Law 1700 and the Agrarian Reform Law 3545 [44] as part of the land redistribution process. Timber concessions predominated throughout the Bolivian Amazon region after the enactment of these laws [45], but shifted to a predominantly collective tenure system towards the end of the 2000s [39]. Approximately 50% of the Bolivian Amazon is now under community ownership by indigenous and *campesino* communities [39]. Indigenous communities have been able to secure vast tracks of indigenous territories; whereas, *campesino* communities could access land by forming a community with at least ten other families. In this way, each community member could gain access to ~500 ha of land [44]. This 500 ha per household criteria was mainly based on the number of *estradas* (paths opened for rubber tapping) that a household is capable of tapping daily (L. Rojas, personal communication, June 6, 2015). We chose to work with *campesino* communities to reduce heterogeneity on the background, livelihood strategy and access to forest resources of community households [3,46]; and because these communities comprise the majority of the rural population in the region (58%) [39] and have a relatively long tradition on using forest products.

Six *campesino* communities were selected for this study (Table 1). Studied communities were selected based on their long-standing engagement in formal timber management. These six communities represent 2.5% of *campesino* communities of the Bolivian Amazon (out of 245, [39]), are 30–130 km distant from one of the two main regional cities, and together comprise an area of 80711 hectares (Table 1). As in the majority of the *Campesino* communities, the forest is internally delimited by community households to enable household-level decision-making to harvest forest resources [47]. However, collective decision-making is needed for logging timber; even in cases in which logging occurs at the household forest-level. The harvest of forest products at the household forest-level allowed us to account for households and their forest as our main sampling unit. We selected 24 households and their forests (2–5 households per community) representing a wide range of Amazon nut harvesting and logging intensities, equivalent to 3.7–36.4% of the household members forming these communities (Table 1). The large variation in the percentage of participating households is largely due to differences on the number of community households among studied communities (11–135, Table 1). Women as household heads represented 20.8% of the participating households. Selected households lived between 50 m and 20 km away from the forest from where they collect Amazon nut, timber and other NTFPs.

**Table 1:** Social and biophysical characteristics of selected *campesino* communities undertaking community timber management plans (CTMPs) in the Bolivian Amazon. FUG = forest user group.

Level	Social and biophysical characteristics	Community name					
		Primero de Mayo	12 de Octubre	Limón	Loma Alta	Puerto Oro	San Antonio
**Community**	Households (#)	19	79	20	135	18	11
	Sampled households	2	4	5	5	4	4
	Timber benefit sharing type*	Individual	Collective	Mostly Collective	Collective	Mostly Individual	Individual
	FUG members	10	29	17	84	17	0
	Nearest city (km)	110	42	122	29.5	72.9	73.6
	Community area (ha)	4943	16378	16137	24604	12583	6067
	Managed forest (ha)	4942.8	2281	16136.7	16300	12582.9	2839.2
	Logging compartment (ha yr^-1^)	204	180–198	435–660	844–907	497–531	182–204
	Cutting Cycle (yrs.)	20	20	20	20	20	20
	First logging (yrs.)	2007	2000	2004	2006	2007	2004
	Logging events up to 2014 (#)	1	6	7	8	6	5
**Household**	*Terra firme* forest (ha)	385.0 ± 63.6	62.3 ± 26.0	394.9 ± 149.4	214.2 ± 150.6	498.8 ± 204.5	265.8 ± 205.9
	Proportion of *terra firme* forest (%)	78.2 ± 13.1	73.2 ± 29.3	87.5 ± 10.5	65.2 ± 31.8	83.1 ± 11.0	82.9 ± 12.5
	Reproductive Amazon nut trees (# ha^-1^)	1.3 ± 0.7	1.2 ± 0.5	0.9 ± 0.3	1.6 ± 0.7	1.4 ± 0.5	1.1 ± 0.6
	Amazon nut availability (Fruits ha^-1^)	160.3 ± 44.5	154.0 ± 91.9	233.3 ± 138.4	140.8 ± 92.5	146.4 ± 108.1	349.3 ± 221.3
	Timber volume available (m^3^ ha^-1^)	13.2 ± 5.4	8.2 ± 3.0	7.0 ± 5.0	6.7 ± 7.8	5.0 ± 3.3	5.4 ± 3.0
	Amazon nut harvesting intensity (% harvested fruits)	51.3 ± 0.4	64.2 ± 3.2	52.0 ± 30.1	43.7 ± 28.9	38.8 ± 22.0	64.5 ± 23.7
	Logged trees (# ha^-1^)	0.4 ± 0.6	2.7 ± 0.9	1.6 ± 0.6	1.7 ± 1.1	1.9 ± 1.8	1.2 ± 0.4
	Logged volume (m^3^ ha^-1^)	0.7 ± 0.9	9.4 ± 1.8	4.4 ± 2.5	9.9 ± 8.3	4.3 ± 5.2	4.1 ± 2.7
	Amazon nut price in 2014 (USD *Barrica*^-1^**)	60.7 ± 2.1	69.5 ± 2.8	59.9 ± 1.7	64.8 ± 9.7	63.9 ± 1.9	57.7 ± 1.7
	Agricultural area opened between 2010–2014 (ha)	2.0 ± 2.8	4.5 ± 1.3	2.6 ± 0.8	3.4 ± 2.1	4.8 ± 0.5	2.6 ± 1.8

* Categories of ‘timber benefit sharing type’ indicate that timber benefits obtained from a household forest were: never shared with other community households (individual); once collectively shared, but not shared with other community households in most recent years (mostly individual); once individual, but collectively shared with other community households in most recent years (mostly collective); and always shared collectively with other community households (collective).

** A *barrica* is the common measurement unit for selling Amazon nut in the Bolivian Amazon. 1 barrica = 69 Kg. (3boxes).

### Data collection

To answer our research questions, we carried out socioeconomic assessments of selected households and biological assessments of the forest to which these households had *de facto* access. Both assessments took place during the first parts of 2014 and 2015. Out of the 24 selected household forests, only three were solely harvested for Amazon nut in a yearly basis, while 18 were logged once under the legal framework of the 1996 Forestry Law over the last 10 years prior to data collection. In order to log timber under a CTMP, the legal framework requires forest users to carry a tree census of the area to be harvested, to plan a road infrastructure to extract trees, and to leave 20% of harvestable trees (i.e., trees >diameter minimum cutting (DMC) ≥50 cm DBH) in the logging compartment as seed trees. During the two years of data collection, twelve household forests underwent some sort of small-scale logging operation, and some abandoned timber from a previous CTMP was extracted from only one household forest. We refer to these two sources of timber income as ‘timber extra CTMP’ in the rest of the manuscript. At the end of this section we describe how we organized the data to answer each one of our research questions, but before this, we describe how we obtained the socioeconomic and biophysical data separately.

#### Socioeconomic assessment

Survey questionnaires from the Poverty and Environment Network (PEN) were adapted into one comprehensive household-level questionnaire for the purpose of this research (S1 File). We collected socioeconomic information from 24 households (Tables 1 and 2). This research did not require approval from the Social Sciences Ethics Committee (SEC) at Wageningen UR because the survey questionnaire did not involve any political, medical or conflict sensitive issues; neither tried to obtain access to traditional knowledge or to other types of knowledge protected by international and national legislations. However, we accounted with the endorsement of the communities’ associations at the regional level (*Federación Sindical Única de Trabajadores Campesinos de Pando* (FSTUCP) in Pando and *Federación Sindical Única de Trabajadores Campesinos Regional Vaca Diez* (FSUTCRVD) in the Vaca Díez province in Beni) and with a research collaboration agreement with each community (S2 File). Such agreement–signed by each community leader–enabled us to carry interviews to voluntary participants. An oral consent of the participating household heads in the survey questionnaires were also requested upon making the voluntary purpose of the survey clear. The questionnaires contained questions that recalled information of the last year (periods: 2013–2014 and 2014–2015), but also held questions that recalled information of the last 5 years (i.e., period 2009–2014). We assumed one-year recalling data as highly accurate given the seasonal allocation of the main production activities spanning a typical production calendar. The survey was conducted amongst the household heads, but often their wives/husbands were also actively involved. Survey questionnaires were carried out after the two weeks in which the vegetation sampling at each community was done. Survey questions were focused on obtaining information of household attributes: social assets, local ecological knowledge, market accessibility, financial assets, and natural and physical assets (Table 2). Variables related to market accessibility were calculated based on coordinates taken within the communities, and the house of the participating household member if outside of the community. Questions also gathered information on the income obtained from timber from the CTMP and from extra CTMP, Amazon nut, other NTFPs, hunting, fishing, agriculture, livestock, salary, business, and gifts. The price at which each household sold a *barrica* of Amazon nut was also recorded. To calculate the yearly timber income that a household obtained from the CTMP, we accounted for the times timber benefits were shared or were individually obtained. Yearly timber income was then deducted from projecting the income obtained so far by a household to the usual 20 years of the timber cutting cycle. For example: in a shared (collective) timber-benefit scenario, a household received three times USD 400 over a 5-year period since the start of the CTMP, thus its yearly timber income was USD 240 [(400 x 3)/5]; whereas, in an individually shared timber-benefit scenario, in which a household derived income from timber once every 20 years (cutting cycle), we divided the amount received from timber over 20. We then separated incomes in three large groups: forest (timber, Amazon nut, other NTFPs and hunting), husbandry (agriculture, agroforestry and livestock) and off-farm income (salary, business and gifts). For all incomes, we calculated the net income as the gross income minus the production costs. Production costs included all monetary costs a household incurred during the production and/or harvest of a specific product (i.e., transport, extra labour, materials and food expenses). Our calculation of production costs does not account for the labour cost of family members. We discriminated subsistence from cash net income and values were converted to US Dollars at the exchange rate of Bs 1 = USD 0.148.

**Table 2:** Socioeconomic and biophysical variables potentially determining income from forests, husbandry, off-farm, Amazon nut and timber at community household forests that were collected in this study.

Attributes	Attribute indicators	Unit of measurement	Explanation
**Social assets**	Household head’s education	Years	Years of formal education
	Residence time	Years	Number of years since a household is using the sampled area of forest
	# of working adults	Number of working adults	Proportion of economically active (working) members in a household
	Position in the community		Position or role occupied by a household head: 0. No role, 1. Secondary role in the community (including community founders), 2. Secondary role in a committee or organization, 3. Leading role (community or regional)
	Times timber benefits were shared	proportion	Number of years timber benefits were shared collectively over the number of years that timber was logged under the community timber management plan (CTMP)
**Local ecological knowledge**	# of other NTFPs harvested	Number	Number of forest products harvested apart from Amazon nut and timber
	# of management practices for Amazon nut	number of management practices per year	Number of management practices carried to enhance Amazon nut production at the sampled forest (max. number of practices is 7): re-opening of nut collection paths, liana cutting, liberation of regeneration, burning of the understory around the tree to facilitate nut collection, wounding of the tree bark, on-purpose protection of regeneration and washing of nuts after harvest
	Degree of involvement in the CTMP		A household's degree of involvement in the community timber management plan (CTMP): 0. No member of the forest user group (FUG)—no involvement in the CTMP, 1. FUG member–involvement in a non-specialized task in the CTMP (e.g., opening of paths for tree inventory), 2. FUG member–involvement in a specialized task in the CTMP (e.g., sawyer)
**Market accessibility**	Distance to the nearest city	Km	Distance from the household house at the community to the nearest market or city
	Travel frequency to the nearest city	Number of times month^-1^	Number of times a household head travels to the nearest city per month
	Bargaining power to sell Amazon nut		Based on the possibility of (a) buyer (s) to offer a better price for Amazon nut (1 = lowest price, 7 = highest price): 1. Unknown dealer; 2. Known dealer, 3. Direct processor
**Natural and physical assets**	Amazon nut fruit production	Fruits ha^-1^	The number of fruits produced per hectare of a household forest
	Timber volume as of 2015	m^3^ ha^-1^	The volume of timber of standing trees > minimum cut diameter (MCD) as of 2015 in a household forest
	Amazon nut harvesting intensity	Percentage	The average percentage of Amazon nut harvested from a household forest over the harvest seasons: 2013–2014 and 2014–2015
	Timber harvesting intensity	m^3^ ha^-1^	The amount of timber harvested from a household forest under the CTMP
	Proportion of *terra firme* forest	Proportional	Proportion of the area of *terra firme* (upland) forest in relation to the land area under household use
	Agricultural area	Hectare	Total area used for shifting cultivation over the last five years
	Value of material assets	USD	Value of all materials and equipment owned by a household
**Financial assets**	Financial support	USD	A household’s total debt to formal institutions, as well as, to informal lenders
	Times external support was received	Number of times in the last 5 years	Number of times a household received support (either technical, in-cash, materials) from external sources over the period of 2009–2014
	Forest income	USD	Total income from forest (subsistence and cash): timber (CTMP and extra CTMP), Amazon nut, other NTFPs and hunting
	Husbandry income	USD	The sum of the net income (cash and subsistence) obtained from slash and burn agriculture, from agroforestry, and from raising domesticated animals (e.g., chicken, pigs, cows)
	Off-farm income	USD	Total income from salary and business earned by a household, in addition to the income from gifts or donations

Based on Uma Shaanker et al. [11] and Duchelle et al. [4]. All variables are measured in a yearly basis, unless specified otherwise.

#### Ecological assessment

**Fig 2:**
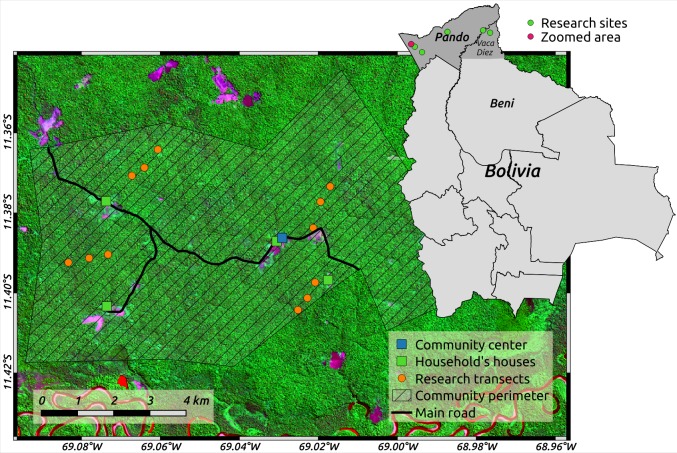
Study site and location of research transects within a community household in the Bolivian Amazon. Image from sentinel 2 satellite (band combination 11/8/2) acquired on August 25, 2016.

We established three 40 m x 500 m (2 ha) transects within each household’s *terra firme* forest to assess the density of Amazon nut trees and of the 17 most commonly harvested timber species (S2 Table). Transects were placed at random distances from each other (varying between 500–1000 m) to comply with sampling independence, and at a random direction to account for the variability on species’ population distribution (Fig 2). All trees ≥10 cm DBH of studied species were inventoried, mapped and tagged in 2014, and re-measured in 2015.

With this information, we calculated timber availability as the volume of standing trees with a diameter >DMC per hectare accounting for species’ differentiated DMC as specified in Bolivia’s forestry rules (Ministerial Resolution 248/1998). We used the formula developed by Metcalf et al. (Formula 1 [48]) to estimate the DBH of buttressed trees and of trees measured at a different measurement height than the standard 1.3 m DBH measurement height. This formula is the most reliable tapering approximation for tropical trees [49].

$$D=\frac{d_{h}}{e^{b_{i}(h-1.3)}}$$ (1)

Where, D is the diameter at 1.3 m height (cm), d is the diameter at height h (cm), h is the height of diameter measurement (m), and b_i_ is the taper parameter (average value of the posterior means calculated for 5 species = -0.04 [48]). Based on this formula, and for consistency in the formula used for calculating timber volume (i.e., Smalian’s formula) of standing trees and logged trees, we calculated the stump diameter at 0.8 m aboveground (own estimation of stump height), and the crown base diameter of standing trees ≥DMC of the 17 study timber species. We discounted the stump height of the estimated commercial height of standing trees to estimate the trunk length necessary for calculating timber volume. We measured stump and crown base diameters, and trunk length (i.e., distance from the stump to the crown base) of logged trees found within the research transects to calculate timber volume of logged trees. We failed to account for the impact of small-scale logging operations because their incidence within the research transects were minimal; they occurred within few transects of three sampled household forests (12.5%), and disturbed only 0.06% of the total sampled area (144 ha).

At the end of two harvest seasons: 2013–2014 and 2014–2015, we counted all fallen Amazon nut fruits within the 30 m radius from the trunk of each producing tree (≥40 cm DBH) found within the transects. Fallen fruits were classified in one of three categories: harvested by people (i.e., machete-opened fruits commonly found gathered near each tree), opened by agouties (*Dasyprocta* spp.) (i.e., the main seed disperser of *Bertholletia*), or unharvested/ unopened (i.e., fruits not found by the collectors or the seed disperser). From Haugaasen et al. [50] we calculated that 6.3% of fruits were removed by agouties beyond the 30 m from where fruits originally fell below a tree crown. This percentage was not considered in our calculations of fruits count because we assumed similar fruit removal rate in all sampled fruit producing trees and household forests. As the counting was done at the end of the harvest season, we believed that the percentage of non-counted fruits, i.e., apart from the 6.3% removed by agouties, was very small. The total number of fruits found within the three transects established at each household forest was averaged and divided by two to obtain the number of fruits produced ha^-1^ year^-1^ at each household. Thus, the average number of fruits produced ha^-1^ from the two harvest seasons was used as a proxy for Amazon nut availability. We calculated the percentage of harvested fruits by people out of the total number of fruits produced per reproductive tree and used the average percentage of harvested fruits over the two years of this study as a proxy for Amazon nut harvesting intensity. We also calculated the density of reproductive (>40 cm DBH) Amazon nut trees per hectare using the data from the transects.

### Data analysis

We first ran a correlation analysis amongst the 26 predictor variables measured. Pairs of variables with a correlation coefficient greater than 0.68 were considered as covariates [51], in which case only one variable was selected to avoid collinearity in subsequent analyses. In this way, we reduced our number of predictor variables to 23, 3–7 per socioeconomic and biophysical attribute associated to household forests (Table 2).

We built generalized linear models (GLMs) to derive the significant (p <0.1), and otherwise, most important predictors from each of the five socioeconomic and biophysical attributes associated to income from forest, husbandry, off-farm, Amazon nut and timber (Table 3). We ran these analyses using the MuMIn package in R [52]. By using the function “dredge” in the model statement, we could simultaneously deal with categorical and continuous predictors (explanatory variables). We also tested the influence of reproductive Amazon nut tree density on each source of income being tested, but this variable was not a significant predictor of any of the sources of income being tested, hence, it was removed from the models. Similarly, we incorporated Amazon nut price amongst the market accessibility attribute variables, but this variable did not have an effect on any of the sources of income being tested, and was also removed from the models. A total of nine structural equation (SEM) models resulted from interspersing the selected factors from the five attributes: one model for forest, three for husbandry, two for off-farm, two for Amazon nut, and one for timber income (Table 3). The number of models per response variable was determined by the number of significant variables in each attribute associated to the response variable because all possible combinations of variables needed to be tested in order to select the best model (Table 3). We used the lavaan and lavaan.survey packages in R [53] to run the SEM models.

**Table 3:** Best predictors of income derived from forest, husbandry, off-farm, Amazon nut and timber. Values correspond to the weights of the Akaike Information Criteria (AIC) of all possible models in which each variable appears. Significance levels: p <0.01***, p <0.05**, p <0.1*, p >0.1^ (most important variable in the absence of a significant predictor per attribute). At least one variable was selected per attribute for each income source.

Attribute	Explanatory variable			Source of Income		
		Forest	Husbandry	Off-farm	Amazon nut	Timber
Social Assets	Household head’s education	0.15	0.15	0.20	0.34	0.16
	Residence time	0.28	0.28	0.22	0.75**	0.29^
	# of working adults	0.21	0.21	0.18	0.21	0.28
	Position in the community	0.17	0.17	0.75**	0.50	0.21
	Times timber benefits were shared	0.55^	0.55^	0.47	0.83**	0.25
Local ecological knowledge	# of other NTFPs harvested	0.10	0.91**	0.48	0.29^	0.18
	# of management practices for Amazon nut production	0.65*	0.33	0.65*	0.22	0.58^
	Degree of participation in the community timber management plan (CTMP)	0.20	0.18	0.18	0.20	0.09
Market accessibility	Distance to the nearest city	0.35	0.12	0.25^	0.92***	0.22
	Bargaining power to sell Amazon nut	0.62*	0.24	0.13	0.14	0.56^
	Travel frequency to the nearest city	0.14	0.26^	0.18	0.21	0.09
Natural and physical assets	Amazon nut availability	0.16	0.58***	0.18	0.58^	0.16
	Timber volume in 2015	0.69*	0.18	0.23	0.32	0.36^
	Amazon nut harvest intensity	0.17	1.00*	0.30	0.34	0.19
	Timber harvesting intensity	0.20	0.12	0.29	0.25	0.17
	Value of material assets	0.16	1.00***	0.64*	0.28	0.19
	Proportion of *terra firme* forest	0.27	0.17	0.15	0.30	0.28
	Agricultural area	0.16	0.25	0.64*	0.22	0.17
Financial Assets	Financial support	0.22	0.23	0.16	0.21	0.16
	Times external support was received	0.17	0.28^	0.24^	0.36	0.17
	Forest income	na	0.16	0.23	na	na
	Husbandry income	0.16	na	0.19	0.13	0.22
	Off-farm income	0.23^	0.19	Na	0.42^	0.24^

#### SEM model construction

We limited the number of predictors in each model to five by selecting the significant or most important variables per attribute and type of income (Table 3) as this was required given our sample size of 24 households [54]. From the models built per response variable, a single best model was selected under the following criteria. First, the model’s p-value (Chi-square) must be greater than 0.05, which is an indicative of goodness of model fit [55,56]. Second, the best model was selected based on the highest Chi-square estimate of the main response variable involved in the model structure because the majority of the models presented a p-value (Chi-square) >0.05. Since our complete “hypothesized” model structure (Fig 1) tested for the five income sources failed to meet the criteria of model fit under the Monte Carlo simulation probability (MCX2) (i.e. needed because of our small sampling size [57]), we decided to modify the hypothesized model structure 1) by removing three fixed pathways that were not significant in all models tested so far, and 2) by removing the three pathways with the lowest standardized coefficient. We opted for option 1 because the difference between both models was minimal in all cases (S3 Table), and in order to balance the contribution of each attribute into the model.

## Results

### The contribution of forest to household net income

**Fig 3:**
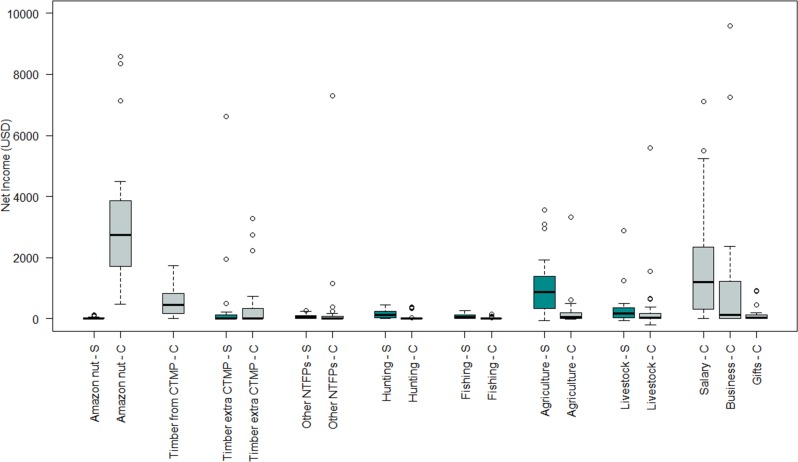
Income of community-based households from different sources by type of income (S) subsistence and (C) cash in the Bolivian Amazon. The upper and lower quartiles in the boxplots explain 25% of the variation in the median net income derived by participating households. Empty circles are the outliers. CTMP = Community timber management plan, NTFPs = Non-timber forest products.

**Fig 4:**
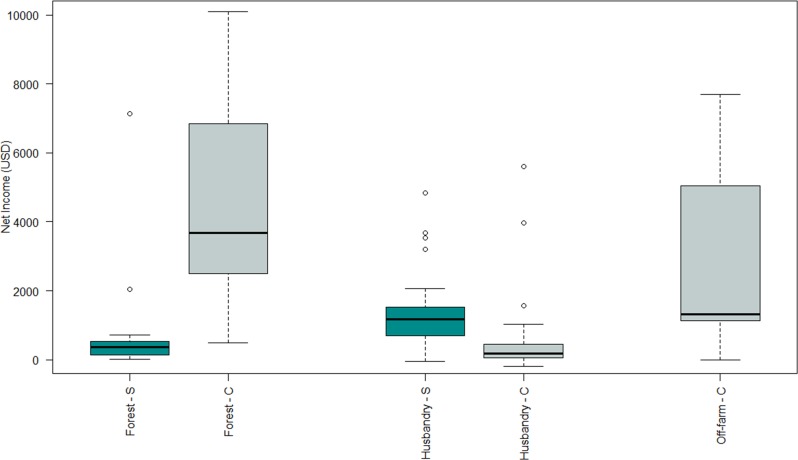
Contribution from forest (timber, Amazon nut, other NTFPs and hunting), husbandry (agriculture, agroforestry and livestock) and off-farm income (salary, business and gifts) incomes to the total net income of community households in the Bolivian Amazon by type of income: (S) subsistence and (C) cash. The upper and lower quartiles in the boxplots explain 25% of the variation in the median net income derived by participating households. Empty circles are the outliers.

A community household in the Bolivian Amazon generates a yearly median net income of USD 9388.51, equivalent to a daily median net income of USD 25.71. Amazon nut alone contributed 44% to a household’s median net income (USD 2811.26), while salary contributed 19% (USD 1191.40), agriculture, 17% (USD 1070.59), timber from the CTMP, 7% (USD 463.15), and timber extra CTMP, 2% (USD 125.80) (S2 Fig). The major contributors to a household’s median cash net income were Amazon nut and salary (86.7% of total cash income); whereas, the majority of a household’s median subsistence net income was derived from agriculture and livestock production (80.2% of total subsistence income; Fig 3). Household heads who logged timber under the CTMP received twice as much timber income than households who did not log timber from their forest and who only benefited from the income shared from the CTMP. Half of the sampled households (n = 12) derived income from timber extra CTMP during the two years of our study, but its relative contribution to the total household income was small (on median 2%). Upon classifying the different sources of household income in three groups (forest, husbandry and off-farm), we found that the overall median contribution of forest reached 59% of a household’s median net income (median = USD 4034.00) (S3 Fig). Nevertheless, community households heavily relied on husbandry income for their subsistence (76%, median = USD 1170.34) (Fig 4).

### Socioeconomic and biophysical factors driving incomes derived from forest, husbandry and off-farm by community households

**Fig 5:**
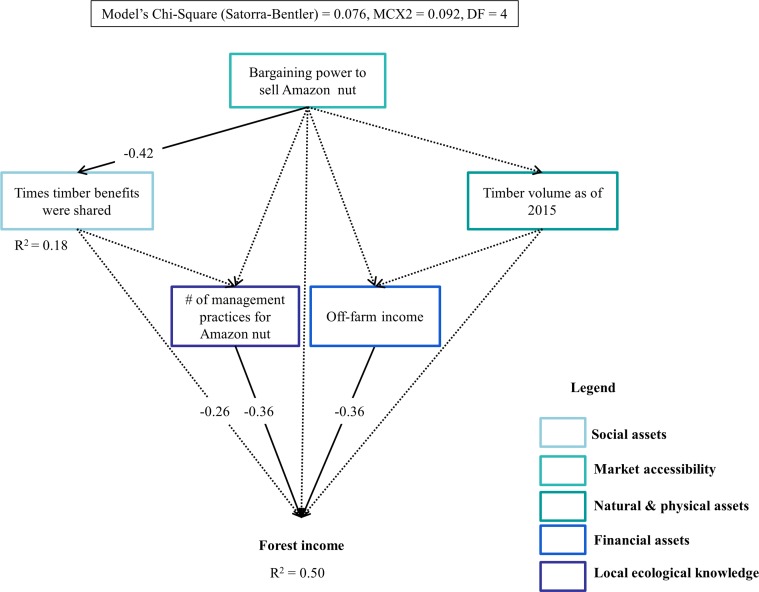
Socioeconomic and biophysical factors determining forest (timber, Amazon nut, other NTFPs and hunting) income of community households in the Bolivian Amazon. Solid arrows indicate significant effects of a variable on another, whereas, dotted arrows indicate non-significant effects. Standardized coefficient values are at the intersection of the arrows indicating the direction of the relationships. Values are only provided for significant relationships that resulted from the structural equation (SEM) models.

**Fig 6:**
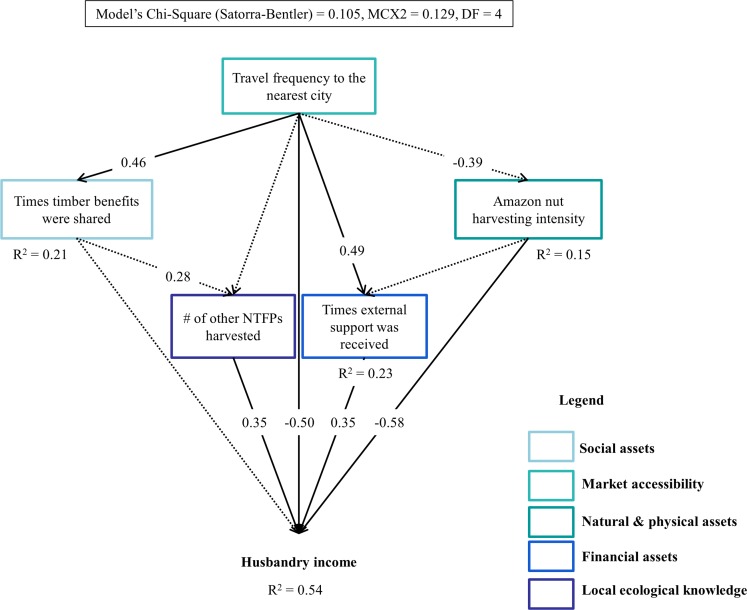
Socioeconomic and biophysical factors determining income derived from husbandry (agriculture, agroforestry and livestock) by community households in the Bolivian Amazon. Solid arrows indicate significant effects of a variable on another, whereas, dotted arrows indicate non-significant effects. Standardized coefficient values are at the intersection of the arrows indicating the direction of the relationships. Values are only provided for significant relationships that resulted from the structural equation (SEM) models.

**Fig 7:**
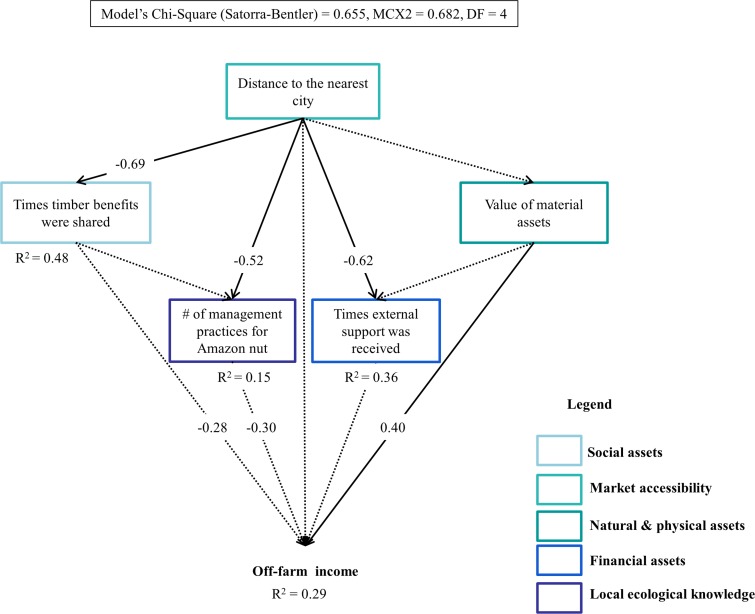
Socioeconomic and biophysical factors determining off-farm (salary, business and gifts) income of community households in the Bolivian Amazon. Solid arrows indicate significant effects of a variable on another, whereas, dotted arrows indicate non-significant effects. Standardized coefficient values are at the intersection of the arrows indicating the direction of the relationships. Values are only provided for significant relationships that resulted from the structural equation (SEM) models.

Results of the SEM analysis indicate that the income that households obtained from the forest decreased as off-farm income (Std coefficient = -0.36) and the number of management practices applied to enhance Amazon nut production (Std coefficient = -0.36) increased (Fig 5). The income derived from husbandry (i.e., agriculture, agroforestry and livestock production) increased with the number of NTFPs being harvested and the external support received by the household (Std coefficient = 0.35 and 0.35, respectively), but, it decreased with the intensity of Amazon nut harvesting and travel frequency of the household head to the nearest market (Std coefficient = -0.58 and -0.50, respectively) (Fig 6). Household heads who travelled more often to the market also received greater external support (Std coefficient = 0.49), increasing further their income from husbandry. Off-farm income only increased as households capitalized on their material assets (Std coefficient = 0.40, Fig 7). No other variable had a significant direct or indirect effect on off-farm income.

### Socioeconomic and biophysical factors driving the income derived from Amazon nut and timber

**Fig 8:**
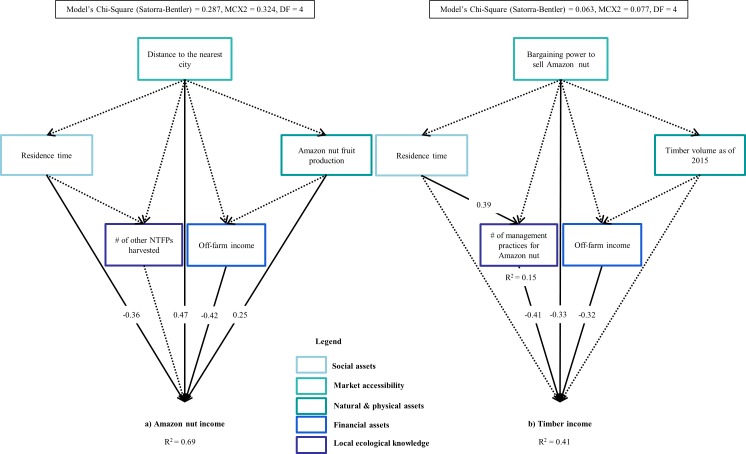
**Socioeconomic and biophysical factors determining the income derived from (a) Amazon nut and (b) timber by community households in the Bolivian Amazon.** Solid arrows indicate significant effects of a variable on another, whereas, dotted arrows indicate non-significant effects. Standardized coefficient values are at the intersection of the arrows indicating the direction of the relationships. Values are only provided for significant relationships that resulted from the structural equation (SEM) models.

The SEM analysis approach also allowed us to find the main socioeconomic determinants of Amazon nut and timber income. Only some of the socioeconomic and biophysical variables that we predicted to determine Amazon nut and timber income had indeed a significant effect on income derived from these forest products. Households further away from the market and with larger Amazon nut availability in their forests derived larger income from Amazon nut (Std coefficient = 0.47 and 0.25, respectively), while households residing for a shorter period of time in the community and relying less on off-farm income also derived a larger income from Amazon nut (Std coefficient = -0.36 and -0.42, respectively) (Fig 8A). Households with a better bargaining power to sell their Amazon nut, those who relied more on off-farm income and applied more management practices to enhance Amazon nut production derived less income from timber (Std coefficient = -0.33, -0.41 and -0.32, respectively) (Fig 8B). Households who resided longer in a community also applied more management practices to enhance Amazon nut production (Std coefficient = 0.39, Fig 8B), decreasing further their income from timber.

## Discussion

### Amazon nut is central to community households’ economy in the Bolivian Amazon

With a share of 44% of the total net income, the Amazon nut is clearly central to the economy of community households in the Bolivian Amazon (S2 Fig). This percentage is comparable to the 45% share of Amazon nut found by Duchelle et al. [4] in 2006–2007, and it is double than the 22% found by Zenteno et al. [3] in 2008–2009. The difference with Zenteno et al.’s study might be due to their focus on the broader regional context comprising the various community configurations as opposed to the more forest-dependent community households of our study. This difference in Amazon nut income may also be due to a higher density of Amazon nut producing trees and timber species at our studied communities who adopted a CTMP, and who also enjoy tenure rights over relatively large tracks of forest [39,47]. Community households at our study site (2014–2015 survey) derived 59% of their income from the forest and 41% from non-forest related activities (i.e., husbandry and off-farm). Our calculated forest income is nearly double than the ~30% of forest income reliance found in two global comparative studies for community-owned forests [29,30]. The degree of dependence over forest, however, falls in between the dependency reported in two previous studies carried out in the region. For example, Duchelle et al. [4] found that community households derived 64% of their overall income from the forest, whereas Zenteno et al. [3] found that community households derived less income from forest (42%). Our findings are more comparable with Duchelle et al.’s in spite that half of their study communities were located inside a wildlife reserve in the Department of Pando where logging is not allowed; whereas, all of our study communities were located outside forest reserves. The close similarity of our results with those of Duchelle et al.’s indicates a strong dependency of *campesino* community households on forests. Given that Duchelle et al.’s study took place a decade earlier, the results of our study may be indicative of a slow decrease in forest income reliance over time, particularly among households who adopted the CTMP.

Income derived from the forest differed largely among studied households (S3 Fig). The largest observed variation among community households was in off-farm income (S3 Fig), which indicates that off-farm income, rather than forest income, is leading to greater income inequality among community households [11,13,29]. Alternatively, forest income dependency may be decreasing due to increasing pressure over forest resources as households become larger, there are more job/business opportunities within the communities, and market accessibility improves [17]. Our calculated yearly household median net income turned out to be 36% higher than the livelihood strategy with the highest median net income found by Zenteno et al [3], i.e., livestock = USD 6000, implying that forest-based livelihoods outperforms livestock-based livelihoods with higher environmental footprint. The price of Amazon nut has doubled from 2009 [3] to 2015 [58], which may–to a large extent–explain the higher forest income obtained by our studied households. Additionally, the long-standing involvement of our studied communities in forest management (i.e., increased net timber income in recent years as a result of the improved legal [58] and structural [17] market accessibility) may explain the higher net income perceived by our studied households. In spite of the increasing total net income of our studied community households, their per capita daily median net income is nearly half of the national daily mean net income: USD 4.28 [a calculation of the daily household’s median net income (USD 25.71) / the median number of household members (6)] vs. USD 8.5 [a calculation of the national gross domestic product for 2014 (USD 3124.1) / 365 days] [59]. We found thus sufficient evidence to affirm that Amazon nut plays a central role on the total income of community households, and that timber income could potentially place community households in a better-off position.

### The role of socioeconomic and biophysical factors on different sources of income derived by community households in the Bolivian Amazon

Attribute indicators of local ecological knowledge and financial assets are the main driving factors of forest and husbandry income, whereas attribute indicators of natural and physical assets determined off-farm income, and to some extent, husbandry income as well (Figs 6 and 7). We predicted that residence time would be the main driving factor of forest income. However, the ability of a household to derive more income from the forest decreased as households applied more management practices to enhance Amazon nut production, which in turn, increased with residence time (Fig 5). This finding is completely unexpected especially because Amazon nut was responsible for the majority of the income derived from the forest. A potential explanation to this might be that households who applied more management practices to increase the production of Amazon nut have less time or are less interested in drawing more income from other forest products such as timber or other commercial NTFPs, decreasing further their income from the forest.

Forest income also decreased as households relied more on off-farm income rather than on husbandry. Off-farm income opportunities demand less work and are more opportunistic than husbandry, and could potentially offset forest income. This means that creating opportunities (e.g., a community forest enterprise or a carpentry) for off-farm income among community households can reduce pressure on forests. However, the implementation of such opportunities needs to go hand in hand with *a priori* knowledge of the pressure these forests can withstand. Even though, market integration (travel frequency of the household head to the market) had a direct negative effect on husbandry income, it also had an indirect positive effect through the times a household received external support (Fig 6). This indicates that households further away from the market are less likely to rely on husbandry income, probably because they base their diet on few agricultural products (i.e., such as manioc, rice and plantain) and go for game hunting instead of raising livestock. These households might also collect other NTFPs more intensively to supplement their diets. In line with our predictions, husbandry income decreased as households harvested Amazon nut more intensively. Farming activities likely keep community households busy, reducing thus the pressure they put on forest resources, mainly over Amazon nut. Our hypothesis that off-farm income will increase with the value of material assets owned by community households is also confirmed, implying that such value might be an indicative of the greater capability of households at obtaining greater off-farm income by investing in business or by undertaking paid jobs [29].

### The role of socioeconomic and biophysical factors on the income derived from Amazon nut and timber by community households in the Bolivian Amazon

Our aim was to identify the socioeconomic and biophysical drivers of the use of Amazon nut and timber at the household level in the Bolivian Amazon. We found that few of the factors we predicted were actually driving Amazon nut income (i.e., off-farm income and Amazon nut availability), and none of our predicted (but other) factors had an effect on timber income. Some of our results contradicted our predictions, particularly when it comes to Amazon nut income (i.e., a positive, rather than a negative influence of distance to the market; and a negative, rather than a positive influence of residence time). Such inconsistencies indicate that certain socioeconomic and biophysical factors determine household incomes in a specific context or scale. Our finding of a positive relationship between Amazon nut income and distance to the market also contradicts our predictions and those of other studies [3] that key forest resource face major pressure closer to the market allowing households to derive a larger income from those resources. The income that households derived from Amazon nut did not depend on their access to better prices (related to closeness to markets), which in turn, did not affect fruit production or the availability of reproductive trees. We also expected that residence time would have a positive influence on Amazon nut income because studies have found that older household heads may dedicate more time to NTFP extraction, yet, the opposite was true among our studied community households, and among community households in Peru and Brazil [4,8] (Fig 8A). The main reason explaining this finding might be that Amazon nut harvesting is rather labour demanding, e.g., it implies carrying approximately 70 kilos over long distances at once. In such case, older residents often give the responsibility of harvesting the Amazon nut from their forest to their offspring, without necessarily receiving a share from the harvesting. This was the case of two, out of the 24 studied households. In addition, and most commonly; sons (dependent and independent household members) living in the city would go to help their parents to harvest Amazon nut because the school holiday season coincides with the Amazon nut production season. These two factors may certainly be adding variation to the data, and are likely the main reasons why we did not find an effect of residence time on Amazon nut income as expected. Although, the number of management practices applied to increase Amazon nut production did not increase Amazon nut income as predicted, liana cutting (a common management practice) alone, could increase fruit production by 77% even 10 years after its application [20].

To our surprise, the degree of involvement in the CTMP was not a significant predictor of the income a household derived from timber, but rather, the number of management practices a household applied to increase Amazon nut production that negatively affected timber income (Fig 8B). A potential explanation for this might be that households tend to carry more management practices to increase their Amazon nut income; and thus, rely less on timber income. We also found that households with better bargaining power to sell their Amazon nut also derived less income from timber (Fig 8B). Thus, we assert that households with greater bargaining power to sell their Amazon nut wait for better Amazon nut prices, and therefore, rely less on timber income. Households with greater bargaining power could potentially derive more income from timber too [10]. For example, we also observed that two studied households were able to increase their earnings by directly offering the sawn timber to a sawmill that offered the best price in the main regional city (Soriano, unpublished data). Chances for households to profit from timber have increased over most recent years with the enactment of the directive 02/2014 that allows the harvest of small-timber volumes for commercial purposes. This is particularly important because around 50% of the studied households could double their income from timber by also harvesting timber extra CTMP during the two year-study period, which was further increased when households harvested timber extra CTMP by themselves. However, we could not test the factors enabling households to incur in this activity because of the few participating households actually performing this activity (4 out of 24 households). We observed, however, that middle-aged household heads–particularly those who had worked at former timber enterprises–were the ones most likely to undertake this activity. Furthermore, off-farm income opportunities could potentially reduce pressure over timber as well because households perceiving greater off-farm income perceived less timber income, independently of the timber available in their forest (Fig 8B). Similarly, high dependence on Amazon nut might decrease a household’s chances of further profiting from timber by devoting more time to carry more management practices to increase Amazon nut production. Finally, some households may choose not to profit from timber yet as they may prefer to keep its timber trees for moments of hardship or sickness [60].

## Conclusions

Hierarchical models such as the SEM modelling approach used in this study helped us disentangle existing inter-relationships among socioeconomic and biophysical factors, which shed light on ways to increase the income derived by community households. Our findings offer insights on how community households can enhance their income, and simultaneously, reduce pressure over keystone forest resources. The modelling approach used for predicting income of *campesino* community households in this study, ie., SEM models, could easily be replicated in other regions, and at varying temporal and spatial scales to come up with sound policy decisions to manage tropical forests accordingly. Even in communities with high degree of reliance on forest income like the communities in the present study, off-farm and husbandry income are complementary to their livelihoods, and can be targeted to improve their living conditions. Although pressure over forest can be overcome by husbandry income, one must be very cautious with the scale of the implementation of husbandry-related activities; particularly, when it turns to cattle ranching expansion [61]. Currently, the majority of the husbandry activities practiced by studied *campesino* communities in the Bolivian Amazon are based on shifting cultivation and raising small livestock (e.g., poultry and pigs). The contribution of cattle ranching is minimal (only four out of 24 households had between 1–17 cows in our sample). These generally “subsistence” driven activities currently practiced by *campesino* community households are certainly being outperformed by Amazon nut and timber production, which may be preventing them from obtaining further economic returns from other sources. For example, a most recent study in the Bolivian Amazon showed a relatively rapid increase of these activities amongst less forest-reliant communities [18].

Given Amazon nut’s importance to community household economies, its highly variable population structure [21], and the continual threat of deforestation [21] for other land uses; multiple-use forest management must be prioritized for the conservation of this rich ecosystem. Considerable external support and research may be required to simultaneously secure a natural resource base and to improve *campesino* community households’ livelihoods over the long run. External support needs to be directed towards capacity building on issues related to multiple-use forest management, and to empower negotiation and investment skills of community households; since these skills allowed them to draw greater income from timber (Fig 8B). Skills they may apply to draw greater income from other forest products as well. Research needs to address the impact of logging and Amazon nut harvesting intensities on *Bertholletia* and timber species populations (Soriano et al., in preparation). Thus, we conclude that the socio-ecological costs of Amazon nut and timber production can be primarily tackled by increasing capacity building on forest management and negotiation and investment skills.

## Supporting information

S1 Fig **Hypothesized socioeconomic and biophysical factors determining the income derived from (a) Amazon nut and (b) timber by community households in the Bolivian Amazon.** A description of the hypothesized factors of the different attributes can be found in Table 2. Solid arrows indicate significant effects of a variable on another, whereas, dotted arrows indicate non-significant effects. AN = Amazon nut, NTFPs = Non-timber forest products, CTMP = Community timber management plan.
(TIF)

S2 Fig Median net income of the different sources of income derived by community households in the Bolivian Amazon. The upper and lower quartiles in the boxplots, each explain 25% of the variation in the median net income derived by participating households. Empty circles are the outliers.
(TIF)

S3 Fig Contribution from forest (timber, Amazon nut, other NTFPs and hunting), husbandry (agriculture, agroforestry and livestock) and off-farm income (salary, business and gifts) incomes to the total net income of community households in the Bolivian Amazon. The upper and lower quartiles in the boxplots explain 25% of the variation in the median net income derived by participating households. Empty circles are the outliers.
(TIF)

S1 Table Studies that combine socioeconomic and biological surveys in their methodological approach to determine the socioeconomic and biophysical drivers of forest resources use. (DOCX)

S2 Table List of timber species sampled in 72 (2 ha) research transects established at community-based household forests in the Bolivian Amazon. These 17 species represent the 10 main timber species harvested in the region according to country-level forestry reports from 2002 to 2012 (ABT, 2002–2012). We ended up with 17 species because the reports only used genera names for several timber species (*Cedrela*, *Dipteryx*, *Hymenaea*, *Tabebuia and Terminalia)*.
(DOCX)

S3 Table Results of the structural equation (SEM) models built for incomes of Amazon nut and timber at community-based household forests. DF = Degrees of freedom.
(DOCX)

S1 File Annual household survey (modified from PEN Questionnaires): Socioeconomic determinants of household wealth and forest use in Bolivian Amazonian communities. Includes Spanish version, the original language in which the survey was carried out.
(DOCX)

S2 File Collaboration agreement signed between the researcher and community leader enabling to carry this research, and consent to interview participating households. Includes Spanish version, the original language in which the agreement was signed upon.
(DOCX)
