# Supplementary figures and images for: Socio-ecological costs of Amazon nut and timber production at community household forests in the Bolivian Amazon
Source: PLoS One. 2017 Feb 24;12(2):e0170594. doi: 10.1371/journal.pone.0170594 (PMC5325212; doi:10.1371/journal.pone.0170594)

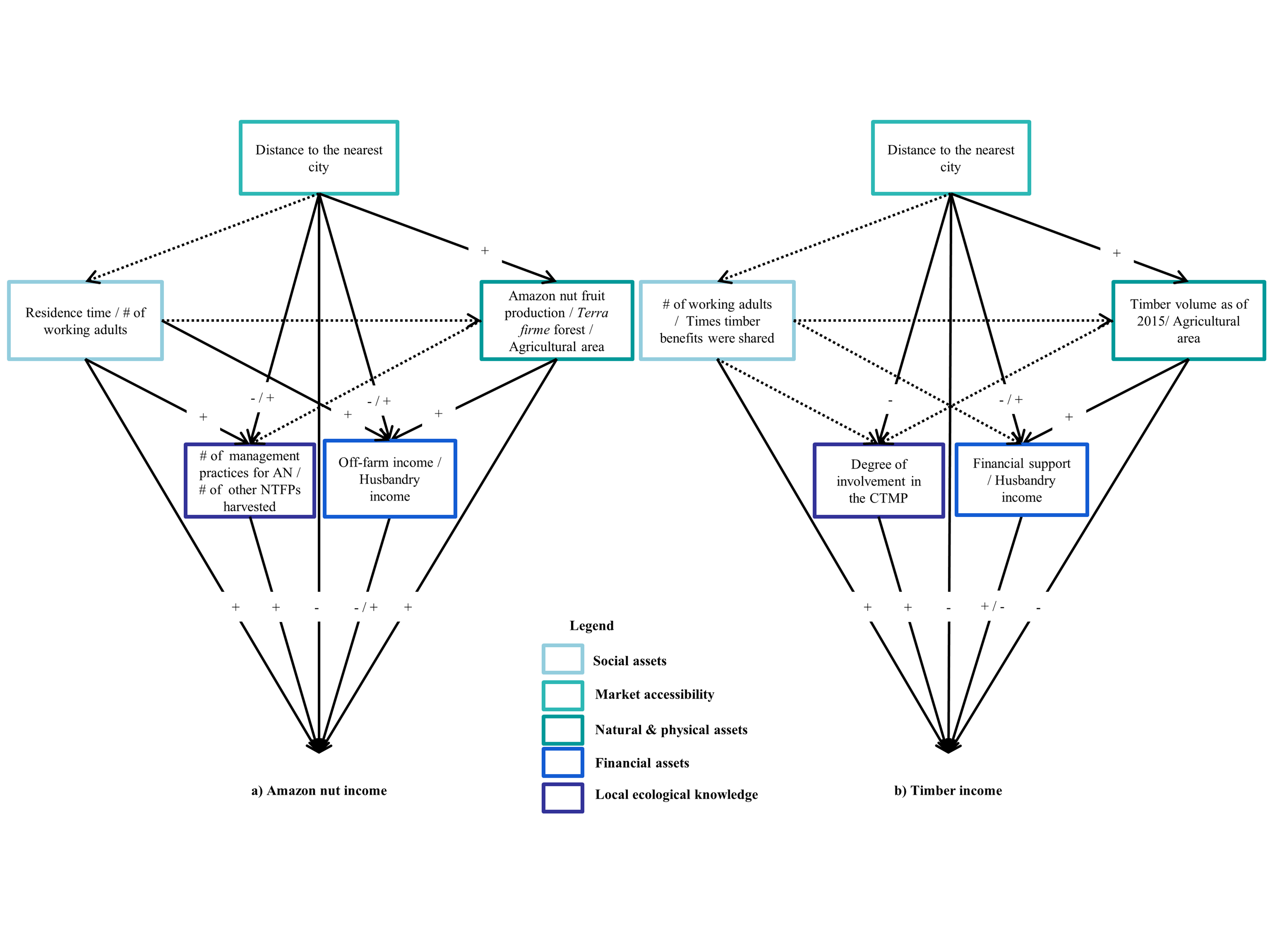

Supplement: S1 Fig — Hypothesized socioeconomic and biophysical factors determining the income derived from (a) Amazon nut and (b) timber by community households in the Bolivian Amazon. A description of the hypothesized factors of the different attributes can be found in Table 2. Solid arrows indicate significant effects of a variable on another, whereas, dotted arrows indicate non-significant effects. AN = Amazon nut, NTFPs = Non-timber forest products, CTMP = Community timber management plan. (TIF) [file pone.0170594.s001.TIF]

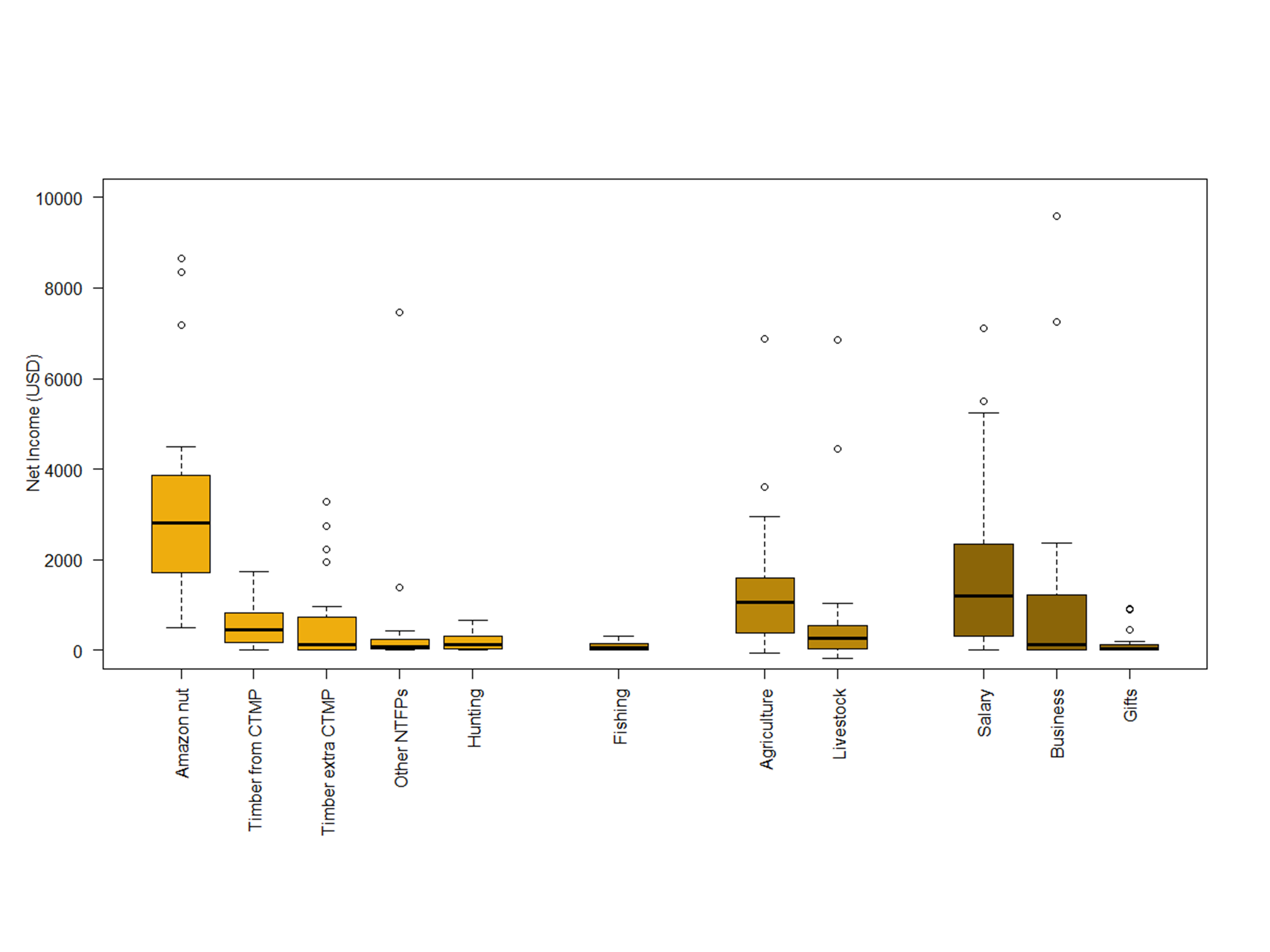

Supplement: S2 Fig — The upper and lower quartiles in the boxplots, each explain 25% of the variation in the median net income derived by participating households. Empty circles are the outliers. (TIF) [file pone.0170594.s002.TIF]

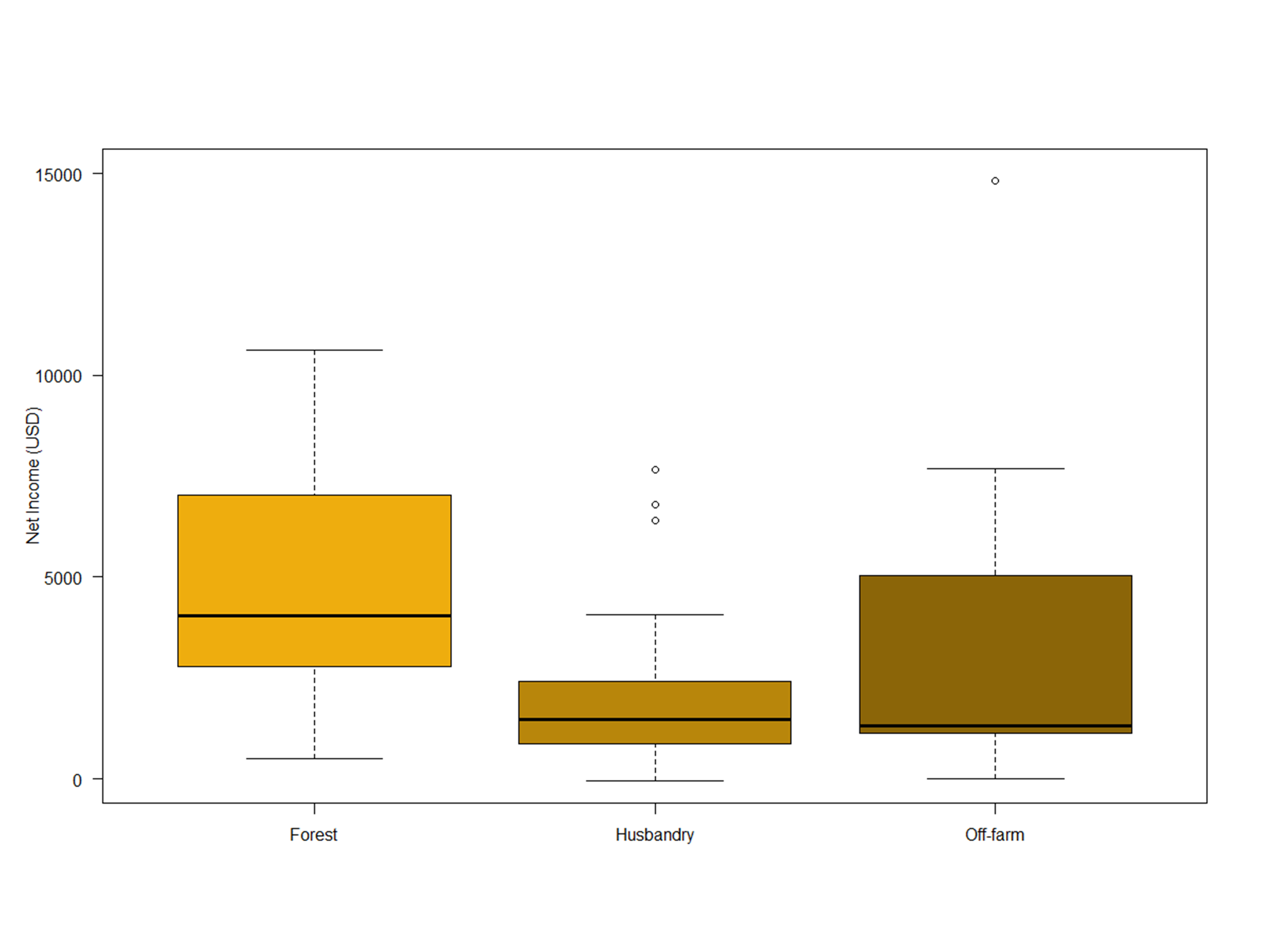

Supplement: S3 Fig — The upper and lower quartiles in the boxplots explain 25% of the variation in the median net income derived by participating households. Empty circles are the outliers. (TIF) [file pone.0170594.s003.TIF]
