# Supplementary material for: Socio-ecological costs of Amazon nut and timber production at community household forests in the Bolivian Amazon
Source: PLoS One. 2017 Feb 24;12(2):e0170594. doi: 10.1371/journal.pone.0170594 (PMC5325212; doi:10.1371/journal.pone.0170594)
Supplement: S1 Table — (DOCX) [file pone.0170594.s004.docx]

**S1 Table.**

| **Author** | **Geographical location** | **Forest products** | **Sampling methods** | | **Analysis approach** |
| --- | --- | --- | --- | --- | --- |
|  |  |  | **Socioeconomic survey** | **Biological survey** |  |
| **Uma Shaanker et al. 2004 (Environmental conservation)** | South India | NTFPs | Three sites: 207 households | Five radiating transects of 1200m at each site | Multiple linear regression |
| **Brown et al. 2011 (PLOS One)** | Ranomafana National Park, Madagascar | Firewood | Eight villages: 247 household questionnaires | Seven (40m x 40m) plots along transects: one in each village | Generalized Linear Models  Regression trees |
| **Mutenje et al. 2011 (Ecological Economics)** | Gonarezhou National Park, Zimbabwe | Firewood | 20 villages: 400 households (20 per village) | Five radiating transects of 10 km per village | Principal Component Analysis (PCA)  Multiple regressions |
| **Zeidemann et al. 2013 (Environmental conservation)** | Central Amazonia, Brazil | Amazon nuts (ANs) | Three sites: 23 households | ANs trails of 6 landholdings, 2500m x 40m transects at non-harvested areas | Univariate models  Generalized linear models |
| **Steele et al. 2014 (Forest Policy and Economics)** | South Africa | Firewood | 8 rural villages: >30 households per village | Three radiating transects of varying lengths (1.8-4.7 km) per village | Principal Component Analysis (PCA)  Multiple stepwise regression |
