# Supplementary material for: Socio-ecological costs of Amazon nut and timber production at community household forests in the Bolivian Amazon
Source: PLoS One. 2017 Feb 24;12(2):e0170594. doi: 10.1371/journal.pone.0170594 (PMC5325212; doi:10.1371/journal.pone.0170594)
Supplement: S2 Table — These 17 species represent the 10 main timber species harvested in the region according to country-level forestry reports from 2002 to 2012 (ABT, 2002–2012). We ended up with 17 species because the reports only used genera names for several timber species (Cedrela, Dipteryx, Hymenaea, Tabebuia and Terminalia). (DOCX) [file pone.0170594.s005.docx]

**S2 Table.**

| Common Name | Species Scientific Name |  |
| --- | --- | --- |
| Almendrillo amarillo | *Apuleia leiocarpa* (J. Vogel) J.F. Macbride | |
| Almendrillo negro | *Dipteryx micrantha* Harms | |
| Cedro fissilis | *Cedrela fissilis* Vell. | |
| Cedro odorata | *Cedrela Odorata* L. | |
| Cuta | *Astronium lecontei* | |
| Mara | *Swietenia macrophylla* King | |
| Mara macho | *Cedrelinga catenaeformis* (Ducke) Ducke | |
| Marfil | *Aspidosperma macrocarpon* C. Martius | |
| Morado | *Peltogyne* cf. *heterophylla* | |
| Paquio | *Hymenaea courbaril* L. | |
| Paquiocillo | *Hymenaea parvifolia* Huber | |
| Roble | *Amburana cearensis* (Allemão) A. C. Smith | |
| Serebo | *Schizolobium parahyba* (Vell. Conc.) S. F. Blake | |
| Tajibo amarillo | *Tabebuia serratifolia* (Vahl) G. Nicholson | |
| Tajibo colorado | *Tabebuia impetiginosa* (C. Martius ex A. DC.) Standley | |
| Verdolago | *Terminalia* spp. | |
| Verdolago amarillo | *Terminalia Oblonga* (Ruíz & Pavón) Steudel | |
