# Supplementary material for: Socio-ecological costs of Amazon nut and timber production at community household forests in the Bolivian Amazon
Source: PLoS One. 2017 Feb 24;12(2):e0170594. doi: 10.1371/journal.pone.0170594 (PMC5325212; doi:10.1371/journal.pone.0170594)
Supplement: S3 Table — DF = Degrees of freedom. (DOCX) [file pone.0170594.s006.docx]

**S3 Table.**

| **Income source** | **Model structure** | **DF** | **Model's p-value (Chi-square)** | **Monte Carlo Simulation probability** | **Main response R^2^** |
| --- | --- | --- | --- | --- | --- |
|  |  |  |  |  |  |
| **Amazon nut** | Model 1. Hypothesized complete model | 1 | 0.243 | 0.000 | 0.65 |
|  | Model 2. Three fixed non-significant pathways removed | 4 | 0.287 | 0.328 | 0.69 |
|  | Model 3. Three least significant pathways removed | 3 | 0.418 | 0.251 | 0.63 |
| **Timber** | Model 1. Hypothesized complete model | 1 | 0.268 | 0.000 | 0.38 |
|  | Model 2. Three fixed non-significant pathways removed | 4 | 0.063 | 0.077 | 0.41 |
|  | Model 3. Three least significant pathways removed | 4 | 0.476 | 0.514 | 0.38 |
| **Forest** | Model 1. Hypothesized complete model | 1 | 0.240 | 0.000 | 0.50 |
|  | Model 2. Three fixed non-significant pathways removed | 4 | 0.076 | 0.092 | 0.50 |
|  | Model 3. Three least significant pathways removed | 4 | 0.332 | 365 | 0.51 |
| **Husbandry** | Model 1. Hypothesized complete model | 1 | 0.093 | 0.000 | 0.57 |
|  | Model 2. Three fixed non-significant pathways removed | 4 | 0.105 | 0.129 | 0.54 |
|  | Model 3. Three least significant pathways removed | 4 | 0.369 | 0.403 | 0.57 |
| **Off-farm** | Model 1. Hypothesized complete model | 1 | 0.526 | 0.000 | 0.32 |
|  | Model 2. Three fixed non-significant pathways removed | 4 | 0.655 | 0.682 | 0.29 |
|  | Model 3. Three least significant pathways removed | 3 | 0.836 | 0.665 | 0.32 |

* Best model structure for Amazon nuts includes significant (z-value <0.05) predictors resulting from the regression model in addition to the hypothesized predictor variables in the absence of significant predictor.
