# Supplementary material for: Socio-ecological costs of Amazon nut and timber production at community household forests in the Bolivian Amazon
Source: PLoS One. 2017 Feb 24;12(2):e0170594. doi: 10.1371/journal.pone.0170594 (PMC5325212; doi:10.1371/journal.pone.0170594)
Supplement: S1 File — Includes Spanish version, the original language in which the survey was carried out. (DOCX) [file pone.0170594.s007.docx]

*With Spanish version below…*

**Annual household survey (modified from PEN Questionnaires): Socioeconomic determinants of household wealth and forest use in Bolivian Amazonian communities**

| **Task** | **Date(s)** | **By who?** | **If not, give comments** |
| --- | --- | --- | --- |
| Interview |  |  |  |
| Checking questionnaire |  |  |  |
| Coding questionnaire |  |  |  |
| Entering data |  |  |  |
| Checking & approving data entry |  |  |  |

1. ***Household identification and general information***

| **Item** | **Name** | **Code** |
| --- | --- | --- |
| Household |  |  |
| Village |  |  |
| Province |  |  |
| Household head |  |  |
| Position/role in the village or other social organization |  |  |
| Country/region of origin |  |  |
| Past occupation |  |  |
| Current occupation |  |  |
| Tenure type |  |  |
| Year of forest access acquisition |  |  |
| Year of tenure right acquisition |  |  |

***B. Household composition***

1. Who are the members of the household?

| **1. Personal Identification number (PID)** | *** Name of household member** | **2. Relation to**  **Household head ^1)^** | **3. Year**  **born**  *(yyyy)* | **4. Sex**  *(0=male*  *1=female)* | **5. Education**  *(number of years completed)* |
| --- | --- | --- | --- | --- | --- |
| 1 |  | Household head = 0 |  |  |  |
| 2 |  |  |  |  |  |
| 3 |  |  |  |  |  |
| 4 |  |  |  |  |  |
| 5 |  |  |  |  |  |
| 6 |  |  |  |  |  |
| 7 |  |  |  |  |  |
| 8 |  |  |  |  |  |
| 9 |  |  |  |  |  |
| 10 |  |  |  |  |  |
| 11 |  |  |  |  |  |
| 12 |  |  |  |  |  |

*1) Codes: 0=household head; 1=spouse (legally married or cohabiting); 2=son/daughter; 3=son/daughter in law; 4=grandchild; 5=mother/father; 6=mother/father in law; 7=brother or sister; 8=brother/sister in law; 9=uncle/aunt; 10=nephew/niece; 11=step/foster child; 12=other family; 13=not related (e.g., servant).*

***C. Geographic location, accessibility and social relations***

| What is the distance from your house to the closest forest limit to which you have access and is utilizable for you? | | 1. … measured in terms of distance | | *km* |
| --- | --- | --- | --- | --- |
|  |  | 2. … measured in terms of time ( by walk/motorcycle/car) | | *Hrs.* |
| What is the main road or river to access to the community and to your house? Describe | | | | |
| Since when there exist a road? | | | *año* | |
| If only road, distance to the nearest commercial town/market | *Km* | | Name the river, if there is one | |
| If not, distance to the nearest road that connects with the nearest commercial town | *Km* | | Indicate mean of transportation | |
| Type of road (asphalted, gravelled, coarse, pathway) |  | | Indicate road type: terciary, secondary, principal (km) | |
| Transport cost in the dry season | *Bs$* | | Price for 1 person | |
| Transport cost in the rainy season | *Bs$* | | Price for 1 person | |
| Frequency |  | | Times in a 3) year, 2) month, 1) week | |
| Stay days in town (Days) | *Days/hours* | | Rainy season | |
|  | *Days/hours* | | Dry season | |
| Could you name the institutions who have supported you over the past 5 years? |  | | Indicate:  institution  Product  Period  Frequency | |
| To what type of buyer did you sell your forest products? |  | | Indicate:  Product &  Type of buyer:  Relative  Known intermediary  Unknown intermediary  Processing plants/sawmill | |

***D. Land use***

1. Please indicate the amount of land (in hectares) that you currently own and have rented in/out.

*Note: See definitions of land categories in the Technical Guidelines.*

| **Category** | **1. Area**  *(ha)* | **2. Ownership**  *(code-tenure)* | **Main products grown/harvested in the past 12 months Max 3. If Brazil nut, indicate # of reproductive trees** *(code-product)* | | |
| --- | --- | --- | --- | --- | --- |
|  |  |  | **3. Rank1** | **4. Rank2** | **5. Rank3** |
| *Forest:* |  |  |  |  |  |
| 1. Natural forest (upland forest) |  |  |  |  |  |
| 2. Natural forest (seasonally flooded, flooded forest) |  |  |  |  |  |
| 3. Forest under some sort of use |  |  |  |  |  |
| 4. Managed forests |  |  |  |  |  |
| 5. Fallow |  |  |  |  |  |
| 6. Plantations |  |  |  |  |  |
|  |  |  |  |  |  |
| *Agricultural land:* |  |  |  |  |  |
| 7. Cropland |  |  |  |  |  |
| 8. Pasture (natural or planted) |  |  |  |  |  |
| 9. Agroforestry |  |  |  |  |  |
| 10. Silvipasture |  |  |  |  |  |
|  |  |  |  |  |  |
| 11. Other vegetation types/land uses (residential, bush, grassland, wetland, etc.) |  |  |  |  |  |
| **12. Total land owned (1+2+3+…+9)** |  |  |  |  |  |
| 13. Land rented out (**included** in 1-9) |  |  |  |  |  |
| 14. Land rented in (**not included** in 1-9) |  |  |  |  |  |

***E. Assets and savings (household wealth)***

1. Please indicate the type of house you have?

| 1. Do you have your own house? ^1)^ |  |
| --- | --- |
| 2. What is the type of material of (most of) the walls? ^2)^ |  |
| 3. What is the type of material of (most of) the roof ? ^3)^ |  |
| 4. How many m^2^ approx. is the house? | m^2^ |

*1) Codes: 0=no; 1=own the house on their own; 2=own the house together with other household(s); 3=renting the house alone; 4=renting the house with other household(s); 9=other, specify:*

*2) Codes: 1=mud/soil; 2=wooden (boards, trunks); 3=iron (or other metal) sheets; 4=bricks or concrete; 5=reeds/straw/grass/fibers/bamboo; 9=other, specify:*

*3) Codes: 1=thatch; 2=wooden (boards); 3=iron or other metal sheets; 4=tiles; 9=other, specify:*

2. Please indicate the number and value of implements and other large household items that are owned by the household.

|  | **1. No. of units owned** | **2. Total value (current sales value of all units, not purchasing price)**  (indicate with “0” if item is not owned) |
| --- | --- | --- |
| 1. Car/truck |  |  |
| 2. Tractor |  |  |
| 3. Motorcycle |  |  |
| 4. Bicycle |  |  |
| 5. Cellphone/phone |  |  |
| 6. TV |  |  |
| 7. Radio |  |  |
| 8. Cassette/CD/ VHS/VCD/DVD/ player |  |  |
| 9. Stove for cooking (gas or electric only) |  |  |
| 10. Refrigerator/freezer |  |  |
| 11. Fishing boat and boat engine |  |  |
| 12. Chainsaw |  |  |
| 13. Plough |  |  |
| 14. Scotch cart |  |  |
| 15. Shotgun/rifle |  |  |
| 16. Energy generator |  |  |
| 17. Wooden cart or wheelbarrow |  |  |
| 18. Water pump |  |  |
| 19. Solar panel |  |  |
| 20. TV antenna |  |  |
| 21. |  |  |
| 22. Others (worth more than approx. 50 USD purchasing price ) |  |  |

3. Please indicate the savings and debt the household has.

| *1.* How much savings does the household have in total? | ***Bs$*** |
| --- | --- |
| - How much does the household have in savings in banks, credit associations or savings clubs? | *Bs$* |
| - How much does the household have saved in loans to family, close relatives, friends? | *Bs$* |
| - How much does the household have in savings in non-productive assets such as gold and jewellery? | *Bs$* |
| - Other, specify (___________________________) | *Bs$* |
| 2. How much does the household have in outstanding debt? | ***Bs$*** |
| - To formal financing entities? | *Bs$* |
| - To family, close relatives, and friends? | *Bs$* |
| - To buyers, intermediaries, wholesalers? | *Bs$* |
| - Other, specify (___________________________) | *Bs$* |

***F. Forest User Groups (FUG)***

*Note: The enumerator should first explain what is meant by a FUG, cf. the Technical Guidelines.*

| 1. Are you or any member of your household a member of a Forest User Group (FUG)? *If ‘no’, go to 11.* | | *(1-0)* |
| --- | --- | --- |
| 2. Does someone in your household normally/regularly attend the FUG meetings? *If ‘no’, go to 5.* | | *(1-0)* |
| 3. **If ‘yes’**: in your household, who normally attends FUG meetings and participates in other FUG activities? *Codes: 1=only the wife; 2=both, but mainly the wife; 3=both participate about equally; 4=both, but* *mainly the husband; 5=only the husband; 6=mainly son(s); 7=mainly daughter(s); 8=mainly* *husband & son(s); 10=mainly wife & daughter(s); 9=other arrangements not described above.* | |  |
| 4. How many person days (= full working days) did the household members spend in total on FUG activities (meetings, policing, joint work, etc) over the past 12 months? | | *days* |
| 5. Does your household make any cash payments/contributions to the FUG? *If ‘no’, go to 7.* | | *(1-0)* |
| 6. **If ’yes’:** how much did you pay in the past 12 months? | | *(Bs$)* |
| 7. Did your household receive any cash payments from the FUG (e.g., share of sales) in the past 12 months? *If ‘no’, go to 9.* | | *(1-0)* |
| 8. **If ‘yes’:** how much did you receive in the past 12 months? | | *(Bs$)* |
| 9. What are your reasons for joining the FUG? *Please rank the most* *important reasons, max 3.* | **Reason** | **Rank 1-3** |
|  | 1. Increased access to forest products |  |
|  | 2. Better forest management and more benefits in future |  |
|  | 3. Access to other benefits, e.g., government support or donor programmes |  |
|  | 4. My duty to protect the forest for the community and the future |  |
|  | 5. Being respected and regarded as a responsible person in village |  |
|  | 6. Social aspect (meeting people, working together, fear of exclusion, etc.)\| |  |
|  | 7. Forced by Government/chiefs/neighbours |  |
|  | 8. Higher price for forest product |  |
|  | 9. Receipt of direct payments |  |
|  | 10. Makes harvest of forest products more efficient |  |
|  | 11. Learn new skills/information |  |
|  | 12. Reduce conflicts over resource |  |
|  | 13. Participation (involvement) in management activities |  |
|  | 14. Other, specify: |  |
| 10. Overall, how would you say the existence of the FUG has affected the benefits that the household gets from the forest? *Codes: 1=large negative effect; 2=small negative effect; 3=no effect; 4=small positive effect;* *5=large positive effect.* | |  |
| 11. **If you don’t participate in FUG,** why? *Please rank the most* *important reasons, max. 3* | **Reason** | **Rank 1-3** |
|  | 1. No FUG exists in the village neither nearby |  |
|  | 2. I’m new in the village/association |  |
|  | 3. FUG members generally belong to other group(s) (ethnic, political party, religion, age, etc.) than I do |  |
|  | 4. Cannot afford to contribute the time |  |
|  | 5. Cannot afford to contribute the required cash payment |  |
|  | 6. FUG membership will restrict my use of the forest, and I want to use the forest as I need it |  |
|  | 7. I don’t believe FUG is very effective in managing the forest |  |
|  | 8. Lack of forest products |  |
|  | 9. Not interested in the activities undertaken by existing FUGs |  |
|  | 10. Corruption in FUG |  |
|  | 11. Interested in joining but needs more information |  |
|  | 12. FUG exists in village/nearby, but household is unaware of its presence |  |
|  | 13. Other, specify: |  |

***G. Crisis and unexpected expenditures***

1. Has the household faced any major income shortfalls or unexpectedly large expenditures during the **past 12 months**?

| **Event** | **How**  **severe?^1)^** | **How did you cope with the income**  **loss or costs?** *Rank max. 3^2)^* | | |
| --- | --- | --- | --- | --- |
|  |  | **2. Rank1** | **3. Rank2** | **4. Rank3** |
| 1. Significant drop of Brazil nut price |  |  |  |  |
| 1. Significant drop of benefits received from GUF |  |  |  |  |
| 1. Serious crop failure |  |  |  |  |
| 1. Serious illness in family (productive age-group adult unable to work for more than one month during past 12 months, due to illness, or to taking care of ill person; or high medical costs) |  |  |  |  |
| 1. Death of productive age-group adult |  |  |  |  |
| 1. Land loss (expropriation, etc.) |  |  |  |  |
| 1. Major livestock loss (theft, drought, etc.) |  |  |  |  |
| 1. Other major asset loss (fire, theft, flood, etc.) |  |  |  |  |
| 1. Lost wage employment |  |  |  |  |
| 1. Wedding or other costly social events |  |  |  |  |
| 1. Payment for sale of hh products arrive later than expected |  |  |  |  |
| 1. Other, specify: |  |  |  |  |

*1) Codes severity: 0=no crisis; 1=yes, moderate crisis; 2=yes, severe crisis. See Technical Guidelines for definitions.*

*2) Codes coping:*

*1. Harvest more forest products*

*2. Harvest more wild products not in the forest*

*3. Harvest more agricultural products*

*4. Spend cash savings*

*5. Sell assets (land, livestock, etc.)*

*6. Do extra casual labour work*

*7. Assistance from friends and relatives*

*8. Assistance from NGO, community org., religious org. or similar*

*9. Get loan from money lender, credit association, bank etc.*

*10. Tried to reduce household spending*

*11. Did nothing in particular*

*12. Spent savings / retirement money*

*13. Reduced number of meals taken*

*14. Borrowed against future earnings*

*15. Sold food that would otherwise be used for household consumption*

*16. Rented out land*

*17. Started new business*

*18. Changed cropping patterns or types of crops planted*

*19. Other, specify:*

*20. Harvested premature crops.*

***H. Forest clearing***

| 1. Did the household clear any forest during the **past 12 months**? *If ‘no’, go to 9.* | | *(1-0)* | | |
| --- | --- | --- | --- | --- |
| **If YES:** | 2. How much forest was cleared? | *ha* | | |
|  | 3. What was the cleared forest (land) used for? *Codes: 1=cropping; 2=tree plantation; 3=pasture; 4=non-agric* *uses (Rank max 3)* | 1.Rank1 | 2.Rank2 | 3.Rank3 |
|  | 4. If used for crops (code ‘1’ in question above), which principal crop was grown? *(code-product) Rank max 3* | 1.Rank1 | 2.Rank2 | 3.Rank3 |
|  | 5. What type of forest did you clear? *(code-forest)* |  | | |
|  | 6. If secondary forest, what was the age of the forest? | *years* | | |
|  | 7. What was the ownership status of the forest cleared? *(code tenure)* |  | | |
|  | 8. How far from the house was the forest cleared located? | *km* | | |
| 9. Has the household over the last *5* years cleared forest? *If ‘no’, go to 11.* | | *1-0* | | |
| 10. **If ‘yes’:** how much forest (approx.) has been cleared over the last 5 years? *Note: This should include the area reported in question 2.* | | *Ha* | | |
| 11. How much land used by the household has over the last 5 years been abandoned (left to convert to natural re-vegetation)? | | *Ha* | | |

***I. Welfare perceptions and social capital***

| 1. All things considered, how satisfied are you with your life over the **past 12 months**? *Codes: 1=very unsatisfied; 2=unsatisfied; 3=neither unsatisfied or satisfied; 4=satisfied; 5=very* *satisfied* | |  |
| --- | --- | --- |
| 2. Has the household’s food production and income over the past 12 months been sufficient to cover what you consider to be the needs of the household? *Codes: 1=no; 2=reasonable (just about sufficient); 3=yes* | |  |
| 3. Compared with other households in the village (or community), how well-off is your household? *Codes: 1=worse-off; 2=about average; 3=better-off* | |  |
| 4. How well-off is your household today compared with the situation **5 years ago?** *Codes: 1=less well-off now; 2=about the same; 3=better off now* *If 1 or 3, go to 5. If 2, go to 6.* | |  |
| 5. **If worse- *or* better-off:** what is the main reason for the change? *Please rank the most* *important responses, max 3.* | **Reason: Change in …** | **Rank 1-3** |
|  | 1. off farm employment |  |
|  | 2. land holding (e.g., bought/sold land, eviction) |  |
|  | 3. forest resources |  |
|  | 4. output prices (forest, agric,…) |  |
|  | 5. outside support (govt., NGO,..) |  |
|  | 6. remittances |  |
|  | 7. cost of living (e.g., high inflation) |  |
|  | 8. war, civil strife, unrest |  |
|  | 9. conflicts in village (non-violent) |  |
|  | 10. change in family situation (e.g. loss of family member/a major bread-winner) |  |
|  | 11. illness |  |
|  | 12. access (e.g. new road,…) |  |
|  | 13. increased/reduced land area for agric. production |  |
|  | 14. started a new business/lost or less business |  |
|  | 15. livestock (gain or loss) |  |
|  | 16. increased regulations |  |
|  | 17. Joined cooperative |  |
|  | 18. Forced to travel for family matters |  |
|  | 19. other (specify): |  |
| 6. Do you consider your village (community) to be a good place to live? *Codes: 1=no; 2=partly; 3=yes* | |  |
| 7. Do you **in general** trust people in the village (community)? *Codes: 1=no; 2=partly, trust some and not others; 3=yes* | |  |
| 8. Can you get help from other people in the village (community) if you are in need, for example, if you need extra money because someone in your family is sick? *Codes: 1=no; 2= can sometimes get help, but not always; 3=yes* | |  |

***J. Direct forest income (income from unprocessed forest products)***

1. What are the quantities and values of raw-material forest products the members of your household collected for both own use and sale over **the past month**?

*Note: Income from plantations is defined as forest income, while agroforestry income is categorized as agric. income (H).*

*Note: The quantities of unprocessed forest products used as inputs in making processed forest products should only be reported in section C, table 2, and not in the table below.*

| **1. Forest product** *(code-product)* | **2. Collected by whom? ^1)^** | **Collected where?** | | **5. Quantity collected (7+8)** | **6. Unit** | **7. Own use (incl. gifts)** | **8. Sold (incl. barter)** | **9. Price per unit** | **10. Type of market** *(code-market)* | **11. Gross value (5*9)** | **12. Transport/ marketing costs (total)** | **13. Purch. Inputs & hired labour** | **14. Net income (11-12-13)** |
| --- | --- | --- | --- | --- | --- | --- | --- | --- | --- | --- | --- | --- | --- |
|  |  | **3. Forest type** *(code-land)* | **4. Ownership** *(code-tenure)* |  |  |  |  |  |  |  |  |  |  |
| **FRUITS** |  |  |  |  |  |  |  |  |  |  |  |  |  |
| Motacú |  |  |  |  |  |  |  |  |  |  |  |  |  |
| Majo |  |  |  |  |  |  |  |  |  |  |  |  |  |
| Asaí |  |  |  |  |  |  |  |  |  |  |  |  |  |
| Palmito |  |  |  |  |  |  |  |  |  |  |  |  |  |
| Chocolate |  |  |  |  |  |  |  |  |  |  |  |  |  |
| Chonta |  |  |  |  |  |  |  |  |  |  |  |  |  |
| Lúcuma |  |  |  |  |  |  |  |  |  |  |  |  |  |
|  |  |  |  |  |  |  |  |  |  |  |  |  |  |
|  |  |  |  |  |  |  |  |  |  |  |  |  |  |
|  |  |  |  |  |  |  |  |  |  |  |  |  |  |
| **SEEDS** |  |  |  |  |  |  |  |  |  |  |  |  |  |
| Castaña |  |  |  |  |  |  |  |  |  |  |  |  |  |
|  |  |  |  |  |  |  |  |  |  |  |  |  |  |
|  |  |  |  |  |  |  |  |  |  |  |  |  |  |
| **LEAVES** |  |  |  |  |  |  |  |  |  |  |  |  |  |
| Jatata |  |  |  |  |  |  |  |  |  |  |  |  |  |
|  |  |  |  |  |  |  |  |  |  |  |  |  |  |
|  |  |  |  |  |  |  |  |  |  |  |  |  |  |
| **BARK** |  |  |  |  |  |  |  |  |  |  |  |  |  |
|  |  |  |  |  |  |  |  |  |  |  |  |  |  |
|  |  |  |  |  |  |  |  |  |  |  |  |  |  |
| **OLEO/LATEX** |  |  |  |  |  |  |  |  |  |  |  |  |  |
| Miel |  |  |  |  |  |  |  |  |  |  |  |  |  |
| Copaibo |  |  |  |  |  |  |  |  |  |  |  |  |  |
| Goma |  |  |  |  |  |  |  |  |  |  |  |  |  |
|  |  |  |  |  |  |  |  |  |  |  |  |  |  |
| **LIANAS** |  |  |  |  |  |  |  |  |  |  |  |  |  |
| Chamairo |  |  |  |  |  |  |  |  |  |  |  |  |  |
| Uña de gato |  |  |  |  |  |  |  |  |  |  |  |  |  |
|  |  |  |  |  |  |  |  |  |  |  |  |  |  |
|  |  |  |  |  |  |  |  |  |  |  |  |  |  |
| **FIREWOOD** |  |  |  |  |  |  |  |  |  |  |  |  |  |
| Isigo |  |  |  |  |  |  |  |  |  |  |  |  |  |
| Caricari |  |  |  |  |  |  |  |  |  |  |  |  |  |
| Blanquillo |  |  |  |  |  |  |  |  |  |  |  |  |  |
| Pacai |  |  |  |  |  |  |  |  |  |  |  |  |  |
|  |  |  |  |  |  |  |  |  |  |  |  |  |  |
|  |  |  |  |  |  |  |  |  |  |  |  |  |  |
| **TIMBER** |  |  |  |  |  |  |  |  |  |  |  |  |  |
| Aliso |  |  |  |  |  |  |  |  |  |  |  |  |  |
| Almendrillo |  |  |  |  |  |  |  |  |  |  |  |  |  |
| Cedro |  |  |  |  |  |  |  |  |  |  |  |  |  |
| Cuta |  |  |  |  |  |  |  |  |  |  |  |  |  |
|  |  |  |  |  |  |  |  |  |  |  |  |  |  |
|  |  |  |  |  |  |  |  |  |  |  |  |  |  |
| **ANIMALS** |  |  |  |  |  |  |  |  |  |  |  |  |  |
| Mono |  |  |  |  | K |  |  |  |  |  |  |  |  |
| Guaso |  |  |  |  | K |  |  |  |  |  |  |  |  |
| Paca |  |  |  |  | K |  |  |  |  |  |  |  |  |
| Jochi |  |  |  |  | K |  |  |  |  |  |  |  |  |
| Chancho |  |  |  |  | K |  |  |  |  |  |  |  |  |
| Taitetú |  |  |  |  | K |  |  |  |  |  |  |  |  |
| Tatu |  |  |  |  | K |  |  |  |  |  |  |  |  |
| Anta |  |  |  |  | K |  |  |  |  |  |  |  |  |
| Pava |  |  |  |  | K |  |  |  |  |  |  |  |  |
|  |  |  |  |  | K |  |  |  |  |  |  |  |  |
|  |  |  |  |  |  |  |  |  |  |  |  |  |  |

1. *Codes: 1=only/mainly by wife and adult female household members; 2=both adult males and adult females participate about equally; 3=only/mainly by the husband and adult male household members; 4=only/mainly by girls (<15 years); 5=only/mainly by boys (<15 years); 6=only/mainly by children (<15 years), and boys and girls participate about equally; 7=all members of household participate equally; 8=none of the above alternatives; 9=person employed by and living with the household.*

2. Do you carry some extra activity to augment your Brazil nut production? If so, which activities do you carry of the list below?

- 1. Clearing of Brazil nut trails
  2. Use of fire underneath reproductive trees to facilitate harvest
  3. Enrichment planting
  4. Clearing around seedlings and saplings
  5. Purposefully protecting seedlings and saplings
  6. Liana cutting
  7. Washing nuts after harvest
  8. *Other*

***K. Forest-derived income (income from processed forest products)***

1. What are the quantities and values of processed forest products that the members of your household produced during **the past 12 months?**

| **1. Product** *(code-product)* | **2. Who in the household did the work? ^1)^** | **3. Quantity produced (5+6)** | **4. Unit** | **5. Own use (incl. gifts)** | **6. Sold (incl. barter)** | **7. Price per unit** | **8. Type of market** *(code-market)* | **9. Gross value (3*7)** | **10. Purchased inputs & hired labour** | **11. Transport/ marketing costs** | **12. Net income excl. costs of forest inputs (9-10-11)** |
| --- | --- | --- | --- | --- | --- | --- | --- | --- | --- | --- | --- |
|  |  |  |  |  |  |  |  |  |  |  |  |
|  |  |  |  |  |  |  |  |  |  |  |  |
|  |  |  |  |  |  |  |  |  |  |  |  |
|  |  |  |  |  |  |  |  |  |  |  |  |
|  |  |  |  |  |  |  |  |  |  |  |  |

*1) Codes: 1=only/mainly by wife and adult female household members; 2=both adult males and adult females participate about equally; 3=only/mainly by the husband and adult male household members; 4=only/mainly by girls (<15 years); 5=only/mainly by boys (<15 years); 6=only/mainly by children (<15 years), and boys and girls participate about equally; 7=all members of household participate equally; 8=none of the above alternatives.*

## L. Non-forest environmental income

1. In addition to forest products and fish included in the previous tables, how much of **other wild products** (e.g., from grasslands, fallows, etc.) did your household collect over **the past 12 months**?

| **1. Type of product** *(code-product)* | **Collected where?** | | **4. Quantity collected (6+7)** | **5. Unit** | **6. Own use (incl. gifts)** | **7. Sold (incl. barter)** | **8. Price per unit** | **9. Gross value (4*8)** | **10. Costs (inputs, hired labor, marketing, etc.)** | **11. Net income (9-10)** |
| --- | --- | --- | --- | --- | --- | --- | --- | --- | --- | --- |
|  | **2. Land type** *(code-land)* | **3. Owner-ship**  *(code-tenure)* |  |  |  |  |  |  |  |  |
|  |  |  |  |  |  |  |  |  |  |  |
|  |  |  |  |  |  |  |  |  |  |  |
|  |  |  |  |  |  |  |  |  |  |  |
|  |  |  |  |  |  |  |  |  |  |  |
|  |  |  |  |  |  |  |  |  |  |  |

*Note: Answers in columns 2 and 3 should be consistent with reported land categories.*

***LL. Wage income***

1. Has any member of the household had paid work over **the past 12 months**?

*Note: One person can be listed more than once for different jobs.*

| **1. Household member (PID)** | **2. Type of work**  *(code-work)* | **3. Days worked past year** | **4. Daily wage rate** | **5. Total wage income (3*4)** |
| --- | --- | --- | --- | --- |
|  |  |  |  |  |
|  |  |  |  |  |
|  |  |  |  |  |
|  |  |  |  |  |

***M. Income from own business (not forest or agriculture)***

1. Are you involved in any type of business, and if so, what are the gross income and costs related to that business over **the past year?**

*Note: If the household is involved in several different types of business, you should fill in one column for each business.*

|  | **1. Business 1** | **2. Business 2** | **3. Business 3** |
| --- | --- | --- | --- |
| 1. What is your type of business?^1)^ |  |  |  |
| **2. Gross income (sales)** |  |  |  |
| **Costs:** |  |  |  |
| 3. Purchased inputs |  |  |  |
| 4. Own non-labour inputs (equivalent market value) |  |  |  |
| 5. Hired labour |  |  |  |
| 6. Transport and marketing cost |  |  |  |
| 7. Capital costs (repair, maintenance, etc.) |  |  |  |
| 8. Other costs |  |  |  |
| **9. Net income (2 - items 3-8)** |  |  |  |
|  |  |  |  |
| 10. Current value of capital stock |  |  |  |

*1) Codes: 1=shop/trade; 2=agric. processing; 3=handicraft; 4=carpentry; 5=other forest based; 6=other skilled labour; 7=transport (car, boat,…); 8=lodging/restaurant; 9=brewing; 10=brick making; 11=landlord/real estate; 12=herbalist/traditional healer/witch doctor; 13=quarrying; 14= contracted work (cleaning/maintenance); 15=renting out equipment; 19=other, specify:*

***N. Income from agriculture – crops***

1. What are the quantities and values of crops that household has harvested during **the past 12 months**?

| **1. Crops** *(code-product)* | **2. Area of production** (*m2)* | **3. Total production (5+6)** | **4. Unit (for production)** | **5.Own use (incl. gifts)** | **6. Sold (incl. barter)** | **7. Price per unit** | **8.Total value (3*7)** |
| --- | --- | --- | --- | --- | --- | --- | --- |
| Rice |  |  |  |  |  |  |  |
| Maize |  |  |  |  |  |  |  |
| Yucca |  |  |  |  |  |  |  |
| Beans |  |  |  |  |  |  |  |
| Plantain |  |  |  |  |  |  |  |
| Banana |  |  |  |  |  |  |  |
| Papaya |  |  |  |  |  |  |  |
| Pineapple |  |  |  |  |  |  |  |
| Watermelon |  |  |  |  |  |  |  |
| Grapefruit |  |  |  |  |  |  |  |
| Orange |  |  |  |  |  |  |  |
| Lemon |  |  |  |  |  |  |  |
| Lima |  |  |  |  |  |  |  |
| Pacai |  |  |  |  |  |  |  |
| Mango |  |  |  |  |  |  |  |
| Coffee |  |  |  |  |  |  |  |
| Coca |  |  |  |  |  |  |  |
| Tropical potato |  |  |  |  |  |  |  |
| Cane |  |  |  |  |  |  |  |
| Copuazu |  |  |  |  |  |  |  |
| Guajaba |  |  |  |  |  |  |  |
| Sweet potato |  |  |  |  |  |  |  |
| Cashew |  |  |  |  |  |  |  |
| Advocado |  |  |  |  |  |  |  |
| Onion |  |  |  |  |  |  |  |
| Lettuce |  |  |  |  |  |  |  |
| Tomato |  |  |  |  |  |  |  |
| Parsley |  |  |  |  |  |  |  |
| Spicy pepper |  |  |  |  |  |  |  |
|  |  |  |  |  |  |  |  |
|  |  |  |  |  |  |  |  |

2. What are the quantities and values of inputs used in crop production over **the past 12 months** (this refers to agricultural cash expenditures)?

*Note: Take into account all the crops in the previous table.*

| **1. Inputs** | **2. Quantity** | **3. Unit** | **4. Price per unit** | **5. Total costs (2*4)** |
| --- | --- | --- | --- | --- |
| 1. Seeds |  |  |  |  |
| 2. Fertilizers |  |  |  |  |
| 3. Pesticides/herbicides |  |  |  |  |
| 4. Manure |  |  |  |  |
| 5. Draught power |  |  |  |  |
| 6. Hired labour |  |  |  |  |
| 7. Hired machinery |  |  |  |  |
| 8. Transport/marketing |  |  |  |  |
| 19. Other, specify: |  |  |  |  |
| a. Bags |  |  |  |  |
| b. Machete |  |  |  |  |
| c. Ax |  |  |  |  |
| d. Lime |  |  |  |  |
| e. Emery |  |  |  |  |
| f. Hoe |  |  |  |  |
| g. Shovel |  |  |  |  |
| h. Fossa |  |  |  |  |
| i. Manual machine for seeding |  |  |  |  |
|  |  |  |  |  |
|  |  |  |  |  |
| 20. Payment for land rental |  |  |  |  |

***O. Income from livestock***

1. What is the number of ADULT animals your household has now, and how many have you sold, bought, slaughtered or lost during **the past 12 months**?

| **1. Livestock** | **2. Beginning number (1 year ago)** | **3. Sold (incl. barter), live or slaughtered** | **4.Slaughtered for own use (or gift given)** | **5. Lost (theft, died,..)** | **6. Bought or gift received** | **7. New from own stock** | **8. End number (now) (2-3-4-5+6+7)** | **9. Price per adult animal** | **10. Total end value (8*9)** |
| --- | --- | --- | --- | --- | --- | --- | --- | --- | --- |
| 1. Cattle |  |  |  |  |  |  |  |  |  |
| Bull |  |  |  |  |  |  |  |  |  |
| Dairy cow |  |  |  |  |  |  |  |  |  |
| Beef cattle |  |  |  |  |  |  |  |  |  |
| 2. Buffalos |  |  |  |  |  |  |  |  |  |
| 3. Goats |  |  |  |  |  |  |  |  |  |
| 4. Sheep |  |  |  |  |  |  |  |  |  |
| 5. Pigs |  |  |  |  |  |  |  |  |  |
| 6. Donkeys |  |  |  |  |  |  |  |  |  |
| 7. Ducks |  |  |  |  |  |  |  |  |  |
| 8. Chicken |  |  |  |  |  |  |  |  |  |
| 9. Horses |  |  |  |  |  |  |  |  |  |
| 10. Rabbit |  |  |  |  |  |  |  |  |  |
| 19. Other, specify: |  |  |  |  |  |  |  |  |  |

2. What are the quantities and values of animal products and services that you have produced during **the past 12 months**?

| **1. Product/service** | **2. Production (4+5)** | **3. Unit** | **4. Own use (incl. gifts)** | **5. Sold (incl. barter)** | **6. Price per unit** | **7. Total value (2*6)** |
| --- | --- | --- | --- | --- | --- | --- |
| 1. Meat ^1)^ |  |  |  |  |  |  |
| 2. Milk ^2)^ |  |  |  |  |  |  |
| 3. Butter |  |  |  |  |  |  |
| 4. Cheese |  |  |  |  |  |  |
| 5. Ghee |  |  |  |  |  |  |
| 6. Eggs |  |  |  |  |  |  |
| 7. Hides and skin |  |  |  |  |  |  |
| 8. Wool |  |  |  |  |  |  |
| 9. Manure |  |  |  |  |  |  |
| 10. Draught power |  |  |  |  |  |  |
| 11. Bee hives |  |  |  |  |  |  |
| 12. Honey |  |  |  |  |  |  |
| 19. Other, specify: |  |  |  |  |  |  |

*1) Make sure this corresponds with the above table on sale and consumption of animals.*

*2) Only milk consumed or sold should be included. If used for making, for example, cheese it should not be reported (only the amount and value of cheese).*

3. What are the quantities and values of inputs used in livestock production during **the past 12 months** (cash expenditures)?

*Note: The key is to get total costs, rather than input units.*

| **1. Inputs** | **2. Unit** | **3. Quantity** | **4. Price per unit** | **5. Total costs (3*4)** |
| --- | --- | --- | --- | --- |
| 1. Feed/fodder |  |  |  |  |
| **Salt** |  |  |  |  |
| **Vitamins** |  |  |  |  |
| **Other:** |  |  |  |  |
| 2. Rental of grazing land |  |  |  |  |
| 3. Medicines, vaccination and other veterinary services |  |  |  |  |
| 4. Costs of maintaining barns, enclosures, pens, etc. |  |  |  |  |
| 5. Hired labour |  |  |  |  |
| 6. Inputs from own farm |  |  |  |  |
| 9. Other, specify: |  |  |  |  |

4. Please indicate approx. share of fodder, either grazed by your animals or brought to the farm by household members.

| **Type of grazing land or source of fodder** | | **3. Approx. share** (%) |
| --- | --- | --- |
| **1. Land type** *(Code-land)* | **2. Ownership** *(Code-tenure)* |  |
|  |  |  |
|  |  |  |
|  |  |  |
|  |  |  |
| Total | | 100% |

***P. Other income sources***

1. Please list any other income that the household has received during **the past 12 months**.

| **1. Type of income** | **2. Total amount received past 12 months** |
| --- | --- |
| 1. Remittances |  |
| 2. Support from government, NGO, organization or similar |  |
| 3. Gifts/support from friends and relatives |  |
| 4. Pension |  |
| 5. Payment for forest services |  |
| 6. Payment for renting out land (if in kind, state the equivalent in cash) |  |
| 7. Compensation from logging or mining company (or similar) |  |
| 8. Payments from FUG |  |
| 9. Other, specify: |  |

***Q. Enumerator/researcher assessment of the household***

*Note: This is to be completed by the enumerator.*

| 1. During the last interview, did the respondent smile or laugh? *Codes: (1) neither laughed nor smiled (somber); (2) only smiled; (3) smiled and laughed; (4) laughed openly and frequently.* |  |
| --- | --- |
| 2. Based on your impression and what you have seen (house, assets, etc.), how well-off do you consider this household to be compared with other households in the village? *Codes: 1=worse-off; 2=about average; 3=better-off* |  |
| 3. How reliable is the information **generally** provided by this household? *Codes: 1=poor; 2=reasonably reliable; 3=very reliable* |  |
| 4. How reliable is the information on **forest collection/use** provided by this household? *Codes: 1=poor; 2=reasonably reliable; 3=very reliable* |  |
| 5. If the forest information is not so reliable (code 1 above), do you think the information provided overestimate or underestimate the actual forest use? *Codes: 1=underestimate; 2=overestimate; 3= no systematic over- or underestimation; 4=don’t* *know.* |  |

**Cuestionario anual de hogares (modificado en base a los cuestionarios del PEN): Factores socioeconómicos que influyen en el bienestar del hogar y uso forestal en comunidades de la Amazonía Boliviana**

| **Tarea** | **Fecha(s)** | **¿Por quién?** | **¿Buen estado? Si no, proporcione comentarios** |
| --- | --- | --- | --- |
| Entrevista |  |  |  |
| Revisión del cuestionario |  |  |  |
| Codificación |  |  |  |
| Ingreso de la información |  |  |  |
| Revisión y aprobación del ingreso de la información |  |  |  |

1. ***Identificación e información general del Hogar***

| **Item** | **Nombre** | **Número de ID** |
| --- | --- | --- |
| Hogar |  |  |
| Comunidad |  |  |
| Provincia |  |  |
| Jefe del hogar |  |  |
| Posición o rol en la comunidad u otra organización social a la que pertenece |  |  |
| Procedencia |  |  |
| Ocupación anterior |  |  |
| Ocupación actual |  |  |
| Tipo de tenencia agraria o forestal |  |  |
| Año de ocupación de hecho |  |  |
| Año de adquisición de tenencia legal |  |  |

## B. Composición del hogar

1. ¿Quiénes son los miembros del hogar?

| **1. Número de identificación personal (NIP)** | *** Nombre del miembro del hogar** | **2. Relación con el jefe del hogar^1)^** | **3. Año de nacimiento** | **4. Sexo** *(0=masculino*  *1=femenino)* | **5. Educación** *(número de años completados)* |
| --- | --- | --- | --- | --- | --- |
| 1 |  | Jefe del Hogar = 0 |  |  |  |
| 2 |  |  |  |  |  |
| 3 |  |  |  |  |  |
| 4 |  |  |  |  |  |
| 5 |  |  |  |  |  |
| 6 |  |  |  |  |  |
| 7 |  |  |  |  |  |
| 8 |  |  |  |  |  |
| 9 |  |  |  |  |  |
| 10 |  |  |  |  |  |
| 11 |  |  |  |  |  |
| 12 |  |  |  |  |  |

*1) Códigos: 0=jefe del hogar; 1=esposa/o; 2 hijo/a; 3=yerno/nuera; 4=nieto; 5=madre/padre; 6=suegro/a; 7=hermano/a; 8=cuñado/a; 9=tío/a; 10=sobrino/a; 11=entenados/hijos adoptivos; 12=otros familiares; 13=no emparentados*

***C. Variables geográficas y de acceso***

| ¿Cuál es la distancia entre la casa y el límite del bosque natural o bajo manejo más cercano al que se tiene acceso y puede ser utilizado? | | 1. … medido en términos de distancia | | *km* |
| --- | --- | --- | --- | --- |
|  |  | 2. … medido en términos de tiempo (minutos a pie) | | *Hrs.* |
| ¿Cuál es el camino o río de acceso principal a la comunidad y a su hogar? Describa | | | | |
| Desde cuando existe acceso a camino? | | | *año* | |
| Si solo hay camino, distancia al pueblo comercial más cercano | *Km* | | nombre del rio, que sirve para transporte en la comunidad, sino nulo | |
| De lo contrario, distancia al camino que conecta con el pueblo comercial más cercano | *Km* | | Indicar medio de transporte | |
| Tipo de camino (asfaltado, ripiado, engravado, precario, senda) |  | | Indicar tipo de camino terciario (km), secundario (km) y principal (km) | |
| Costo total de transporte en época seca | *Bs$* | | Precio de 1 persona | |
| Costo total de transporte en época de lluvia | *Bs$* | | Precio de 1 persona | |
| Frecuencia |  | | 3) veces al año, 2) mes, 1) semana | |
| Tiempo de estadía (Días) | *Días/horas* | | Época de lluvia | |
|  | *Días/horas* | | Época seca | |
| Podría nombrar las instituciones de las que recibió apoyo en los últimos 5 años? |  | | Indicar:  institución  producto  Periodo  Frecuencia | |
| A qué tipo de comprador vendió sus productos que extrajo de su bosque? |  | | Indicar:  Producto &  Tipo de comprador:  Pariente  Intermediario conocido  Intermediario desconocido  Procesadores directos/aserradero | |

## D. Uso de la tierra

1. Por favor indicar la cantidad de tierra (en hectáreas) que actualmente posee, ha arrendado o dado en arrendamiento
*Nota: Ver definiciones de las categorías de tierra en las Guías Técnicas.*

| **Categoría** | **1. Área** *(Has.)* | **2. Tenencia** *(código –tenencia)* | **Principales cultivos producidos y cosechados en los últimos 12 meses Max. 3. Si castaña, indicar # árboles reproductivos** *(código-producto)* | | | | |
| --- | --- | --- | --- | --- | --- | --- | --- |
|  |  |  | **3. Rango1** | **4. Rango2** | | **5. Rango3** | |
| *Bosque:* | | | | | | | |
| 1. Bosque natural (monte alto) |  |  |  | |  | |  |
| 1. Bosque natural (monte bajo o bajura) |  |  |  | |  | |  |
| 1. Bosque bajo algún tipo de uso |  |  |  | |  | |  |
| 1. Bosque bajo manejo |  |  |  | |  | |  |
| 1. Barbecho |  |  |  | |  | |  |
| 1. Plantaciones |  |  |  | |  | |  |
|  | | | | | | | |
| *Tierra agrícola:* | | | | | | | |
| 1. Cultivos |  |  |  | |  | |  |
| 1. Pastos (naturales o plantados) |  |  |  | |  | |  |
| 1. Agroforestería (incluye frutales) |  |  |  | |  | |  |
| 1. Silvopastoreo |  |  |  | |  | |  |
|  |  |  |  | |  | |  |
| 1. Otros tipos de vegetación /usos de la tierra (residencial, arbustos, pantanos, etc.) |  |  |  | |  | |  |
| 1. **Total de tierra en propiedad (1+2+3+…+12)** |  |  |  | |  | |  |
| 1. Tierra dada en arrendamiento (incluida 1-12) |  |  |  | |  | |  |
| 1. Tierra arrendada (no incluida en 1-12) |  |  |  | |  | |  |

## E. Activos y ahorros

1. Por favor indicar el tipo de casa que tiene

| 1. ¿Tiene casa propia? ^1)^ |  |
| --- | --- |
| 2. ¿De qué tipo de material están hechas (la mayoría de) las paredes? ^2)^ |  |
| 3. ¿De qué tipo de material está hecho (la mayor parte de) el techo?^3)^ |  |
| 4. ¿Cuántos m^2^ aproximadamente tiene la casa? | *m^2^* |

*1) Códigos: 0=no; 1=casa propia; 2=casa propia compartida con otro(s) hogar(es); 3=casa alquilada; 4=casa alquilada con otro(s) hogar (es); 9=otro, especificar:*

*2) Códigos: 1=adobe; 2=madera; 3=lámina; 4=ladrillos o concreto; 9=otro, especificar:*

*3) Códigos: 1=paja; 2=madera; 3= calamina; 4=tejas; 9=otros, especificar:*

2. Por favor indicar el número y valor de los implementos y objetos que posee el hogar

|  | **1. No. De unidades** | **2. Valor total (valor actual de venta de todas las unidades, no el precio original de compra)** *(Q, si el objeto no es propiedad del hogar colocar ‘0’)* |
| --- | --- | --- |
| 1. Vehículo |  |  |
| 1. Tractor |  |  |
| 1. Motocicleta |  |  |
| 1. Bicicleta |  |  |
| 1. Teléfono |  |  |
| 1. TV |  |  |
| 1. Radio |  |  |
| 1. Reproductor de Cassette/CD/ VHS/VCD/DVD |  |  |
| 1. Estufa para cocinar (de gas o eléctrica únicamente) |  |  |
| 1. Refrigerador |  |  |
| 1. Lancha pesquera (casco) y motor |  |  |
| 1. Motosierra |  |  |
| 1. Arado |  |  |
| 1. Carreta |  |  |
| 1. Arma de fuego |  |  |
| 1. Motor de luz |  |  |
| 1. Máquina de coser |  |  |
| 1. Bomba de agua |  |  |
| 1. Panel solar |  |  |
| 1. Antena de TV |  |  |
|  |  |  |
| 1. Otros (cuyo precio de compra sea de más que 50 USD ) |  |  |

3. Por favor indicar los ahorros y las deudas que tiene el hogar

| 1. ¿Cuánto tiene el hogar ahorrado en total? | *Bs$* |
| --- | --- |
| - En bancos, asociaciones de crédito o grupos de ahorro | *Bs$* |
| - En préstamos a amigos y parientes | *Bs$* |
| - En activos no productivos como oro y joyas | *Bs$* |
| - Otros, especificar (__________________________) | *Bs$* |
| 1. ¿Cuál es la deuda total del hogar? | *Bs$* |
| - A entidades financieras legalmente establecidas | *Bs$* |
| - A Parientes, compadres y amigos | *Bs$* |
| - A compradores de productos del bosque | *Bs$* |
| - Otros, especificar (__________________________) | *Bs$* |

## F. Grupo de usuarios forestales (GUF)

Nota: El encuestador debe explicar primero que son los GUF, ver Guías Técnicas.

| 1. ¿Pertenece usted o algún miembro de su hogar a un grupo de usuarios forestales (GUF)?  *Si la respuesta es ‘no’, pase a 11* | | *(1-0)* |
| --- | --- | --- |
| 1. ¿Alguien en su hogar asiste regularmente a las reuniones del GUF?  *Si la respuesta es ‘no’, pase a 5* | | *(1-0)* |
| 1. **Si la respuesta es ‘sí’**: en su hogar, ¿quién asiste normalmente a las reuniones del GUF y participa en otras actividades organizadas por este grupo?  *Códigos: 1=sólo la esposa; 2=ambos, pero principalmente la esposa; 3=ambos participan de la misma forma; 4=ambos, pero principalmente el esposo; 5=sólo el esposo; 9=otros arreglos no descritos anteriormente* | |  |
| 1. ¿Cuántos jornales dedicaron los miembros del hogar a actividades del GUF (reuniones, patrullaje, trabajo conjunto, etc.) durante los últimos 12 meses? | | *días* |
| 1. ¿Su hogar contribuye con el GUF haciendo algún pago/contribución en efectivo?   *Si la respuesta es ‘no’, pase a 7* | | *(1-0)* |
| 1. **Si la respuesta es ’sí’:** ¿Cuánto pagó en los últimos 12 meses? (Q) | |  |
| 1. ¿Su hogar recibió algún pago en efectivo por parte del GUF (ej. porcentaje de ventas) en los últimos 12 meses? *Si la respuesta es ‘no’, pase a 9* | | *(1-0)* |
| 1. **Si la respuesta es ‘sí’:** ¿Cuánto recibió en los últimos 12 meses? *(Q)* | |  |
| 1. ¿Cuáles fueron sus razones para integrarse al GUF? *Por favor, señale en orden de prioridad, las razones más importantes. Max 3.* | **Razón** | **Rango 1-3** |
|  | 1. Aumento en el acceso a productos forestales |  |
|  | 1. Mejor manejo forestal y más beneficios para el futuro |  |
|  | 1. Acceso a otros beneficios. Ej. apoyo gubernamental o de la cooperación internacional |  |
|  | 1. Mi obligación de proteger el bosque para la comunidad y el futuro |  |
|  | 1. Ser respetado y reconocido como una persona responsable en la comunidad |  |
|  | 1. Aspectos sociales (conocer gente, trabajar colectivamente, temor de ser excluido, etc.) |  |
|  | 1. Obligado por el Gobierno/líderes locales/vecinos |  |
|  | 1. Obtener mejores precios |  |
|  | 1. Implica la cosecha de los productos más eficientes |  |
|  | 1. Aprender nuevas habilidades e información |  |
|  | 1. Reducir conflictos sobre los recursos |  |
|  | 1. Participar en actividades del manejo |  |
|  | 13. Otro, especificar: |  |
| 1. De forma general, ¿cómo diría usted que la existencia del GUF ha afectado los beneficios que el hogar obtiene del bosque? *Códigos: 1=efecto negativo grande; 2=efecto negativo pequeño; 3=ningún efecto; 4=efecto positivo pequeño; 5=efecto positivo grande.* | |  |
| 1. **Si usted no participa en el GUF,** ¿por qué?  *Por favor, señale el orden de prioridad de las razones más importantes. Max. 3* | **Razón** | **Rango 1-3** |
|  | 1. No existe ningún GUF en esta comunidad |  |
|  | 1. Soy nuevo en la comunidad |  |
|  | 1. Los miembros del GUF pertenecen generalmente a otros grupos (étnicos, políticos, religiosos, etc.) |  |
|  | 1. No tengo el tiempo suficiente |  |
|  | 1. No puedo contribuir con el pago requerido |  |
|  | 1. Mi membresía al GUF restringiría mi uso del bosque, y yo quiero usarlo de acuerdo a mis necesidades |  |
|  | 1. No creo que el GUF sea una forma muy efectiva de manejar el bosque |  |
|  | 1. No hay productos forestales que justifiquen un GUF |  |
|  | 1. No me interesa participar de las actividades que realizan GUF’s existentes |  |
|  | 1. Existe corrupción en el GUF |  |
|  | 1. Le interesa participar pero necesita más información |  |
|  | 1. Hay GUFs en la comunidad pero desconoce su existencia |  |
|  | 1. Otro, especificar: |  |

## G. Crisis y gastos inesperados

¿El hogar ha enfrentado alguna escasez significativa de ingresos o grandes gastos inesperados, durante **el** **último año**?

| **Acontecimiento** | **1. Código^1)^** | **¿Cómo enfrentó las pérdidas o los costos?** *Señalar en orden de prioridad. Max. 3^2)^* | | |
| --- | --- | --- | --- | --- |
|  |  | **3.Rango1** | **4.Rango2** | **5.Rango3** |
| 1. Disminución significativa del precio de la castaña |  |  |  |  |
| 1. Disminución del pago de beneficios del GUF |  |  |  |  |
| 1. Pérdida seria de cultivos |  |  |  |  |
| 1. Enfermedad seria en la familia (adultos económicamente activos, o cuidado de un enfermo, o a altos costos médicos) |  |  |  |  |
| 1. Muerte de un adulto económicamente activo |  |  |  |  |
| 1. Pérdida de tierra (expropiación, etc.) |  |  |  |  |
| 1. Pérdida grande de ganado (robo, sequía, etc.) |  |  |  |  |
| 1. Pérdida grande de otros activos (fuego, robo, inundación, etc.) |  |  |  |  |
| 1. Pérdida de empleo |  |  |  |  |
| 1. Boda u otros eventos sociales costosos |  |  |  |  |
| 1. Retraso en el pago por la venta de productos |  |  |  |  |
| 1. Otro, especificar: |  |  |  |  |

*1) Códigos de severidad de crisis: 0=no; 1=sí, crisis moderada; 2=sí, crisis severa. Ver Guías Técnicas para las definiciones.*

*2) Códigos:*

1. *Cosechar más productos forestales*
2. *Cosechar más productos silvestres fuera del bosque*
3. *Cosechar más productos agrícolas*
4. *Tomar previsiones (ejm. hacer chaco tanto en bajío como monte alto)*
5. *Gastar más ahorros en efectivo*
6. *Venta de activos (tierra, ganado, etc.)*
7. *Trabajo extra ocasional*
8. *Ayuda de amigos y parientes*
9. *Ayuda de ONGs, organizaciones comunales, religiosas o similares*
10. *Obtención de préstamo de un prestamista, asociación de crédito, banco, etc.*
11. *Tratar de reducir el gasto en el hogar*
12. *No se hizo nada en particular*
13. *Se gastó ahorros*
14. *Se redujo la cantidad en la alimentación*
15. *Se prestó a cuenta de futuras cosechas*
16. *Alquiló tierra*
17. *Empezó un nuevo negocio*
18. *Cambió de estrategia de cultivación o tipo de cultivo*
19. *Otro, especificar:*
20. *Cosechó prematuramente*

## H. Tumba del bosque

| 1. ¿El hogar tumbó algo de bosque o barbecho durante el último año?  *Si la respuesta es ‘no’, pase a 9* | | *(1-0)* | | |
| --- | --- | --- | --- | --- |
| **Si la respuesta es *‘si’*:** | 1. ¿Cuánto bosque fue tumbado? | *ha* | | |
|  | 1. ¿Para qué se usó la tierra desmontada? *Códigos: 1=cultivos; 2=plantación de árboles; 3=pastos; 4=usos no agrícolas (Señalar orden de prioridad. Max. 3)* | 1.Rango1 | 2.Rango2 | 3.Rango3 |
|  | 1. Si se usó para cultivos (código ‘1’ en la pregunta anterior), ¿cuál fue el principal cultivo establecido? *(código-producto) Señale orden de prioridad. Max. 3* | 1.Rango1 | 2.Rango2 | 3.Rango3 |
|  | 1. ¿Qué tipo de bosque tumbó? *(código-bosque)* |  | | |
|  | 1. Si era barbecho, ¿qué edad tenía el barbecho? | *años* | | |
|  | 1. ¿Cuál era el tipo de tenencia del bosque tumbado?  *(código tenencia)* |  | | |
|  | 1. ¿A qué distancia de la casa estaba el bosque tumbado? | *km* | | |
| 1. ¿El hogar ha tumbado bosques durante los últimos cinco años?   *Si la respuesta es ‘no’, pase a 11* | | *1-0* | | |
| 1. **Si la respuesta es ‘sí’:** ¿Aproximadamente, cuánto bosque ha sido tumbado durante los últimos cinco años?   *Nota: Esto debería incluir el área reportada en la pregunta 2.* | | *ha* | | |
| 1. ¿Cuánta tierra usada por el hogar ha sido abandonada durante los últimos cinco años (dejada para la regeneración natural de su vegetación)? | | *ha* | | |

## I. Percepción de bienestar y capital social

| 1. Considerando todas las cosas, ¿qué tan satisfecho está usted con su vida durante los últimos 12 meses? *Códigos: 1=muy insatisfecho; 2=insatisfecho; 3=ni insatisfecho ni satisfecho; 4=satisfecho; 5=muy satisfecho* | |  |
| --- | --- | --- |
| 1. ¿Han sido los ingresos del hogar y la producción de comida suficientes durante los últimos 12 meses para cubrir las que usted considera como las necesidades del hogar?  *Códigos: 1=no; 2=razonable (justo lo suficiente); 3=sí* | |  |
| 1. En comparación con otros hogares en la aldea (comunidad), ¿qué tan bien está su hogar? *Códigos: 1=peor; 2=alrededor del promedio; 3=mejor* | |  |
| 1. ¿Qué tan bien está su hogar actualmente en comparación con su situación de **hace 5 años**? *Códigos: 1=peor ahora; 2=casi la misma; 3=mejor ahora  Si 1 ó 3, ir a 5. Si 2, ir a 6.* | |  |
| 1. **Si está peor o mejor** ,¿cuál es la principal razón del cambio? *Por favor asigne un orden de prioridad a las respuestas más importantes , max 3.* | **Razón: Cambio en …** | **Rango 1-3** |
|  | 1. empleo fuera de la parcela |  |
|  | 1. extensión de la parcela (p.ej., tierra vendida/comprada) |  |
|  | 1. recursos forestales |  |
|  | 1. precios de la producción (forestal, agrícola,…) |  |
|  | 1. apoyo externo (gobierno, ONG,…) |  |
|  | 1. remesas |  |
|  | 1. costo de vida (p.ej.,inflación alta) |  |
|  | 1. guerra, conflicto civil, intranquilidad |  |
|  | 1. conflictos en la aldea (no violentos) |  |
|  | 1. situación familiar (p. ej., pérdida de un miembro de la familia que contribuía al sustento) |  |
|  | 1. enfermedad |  |
|  | 1. acceso (p.ej., camino nuevo,…) |  |
|  | 1. aumento/reducción en el área destinada para la producción agr. |  |
|  | 1. empezó/abandonó un negocio |  |
|  | 1. perdió/ganó en producción de animales |  |
|  | 1. mayor regulación |  |
|  | 1. se unió a una asociación/cooperativa |  |
|  | 1. Se vio forzado a viajar por asuntos familiares |  |
|  | 19. otros (especificar): |  |
| 1. ¿Considera usted que su aldea (comunidad) es un buen lugar para vivir?  *Códigos: 1=no; 2=parcialmente; 3=sí* | |  |
| 1. ¿Confía usted **en general** en la gente de su aldea (comunidad)? *Códigos: 1=no; 2=parcialmente, confío en unos y no en otros; 3=sí* | |  |
| 1. ¿Puede usted obtener ayuda de otra gente en su aldea (comunidad) en caso de necesidad, por ejemplo si necesita dinero extra por la enfermedad de un miembro de su familia?  *Códigos: 1=no; 2= a veces puedo obtener la ayuda pero no siempre; 3=sí, cómo?* _________________________________________________________________________________ | |  |

## J. Ingresos directos del bosque (ingreso de productos forestales no procesados)

1. ¿Cuáles son las cantidades y los valores de la materia prima recolectada del bosque por los miembros de su hogar o por externos, tanto para uso doméstico como para la venta durante **el último año**?

*Nota: Las respuestas en las columnas 3 y 4 deben ser consistentes con las categorías de la tierra reportadas*

| **1.**  **Producto forestal** *(código-producto)* | **2. ¿Por quién fue recolectado?^1)^** | **¿Dónde fue recolectado?** | | **5. Cantidad colectada**  **(7+8)** | **6.**  **Unidad** | **7. Uso doméstico (incl. regalos)** | **8.**  **Venta (incl. Intercambio o trueque)** | **9. Precio por unidad** | **10. Tipo de mercado**  *(código-mercado)* | **11. Valor bruto**  **(5*9)** | **12.**  **Costos de transporte y comercialización**  **(total)** | **13. Compra de insumos y pago de mano de obra** | **14.**  **Ingreso neto**  **(11-12-13)** |
| --- | --- | --- | --- | --- | --- | --- | --- | --- | --- | --- | --- | --- | --- |
|  |  | **3. Tipo de tierra**  *(código-tierra)* | **4. Tenencia**  *(código-tenencia)* |  |  |  |  |  |  |  |  |  |  |
| **FRUTOS** |  |  |  |  |  |  |  |  |  |  |  |  |  |
| Motacú |  |  |  |  |  |  |  |  |  |  |  |  |  |
| Majo |  |  |  |  |  |  |  |  |  |  |  |  |  |
| Asaí |  |  |  |  |  |  |  |  |  |  |  |  |  |
| Palmito |  |  |  |  |  |  |  |  |  |  |  |  |  |
| Chocolate |  |  |  |  |  |  |  |  |  |  |  |  |  |
| Chonta |  |  |  |  |  |  |  |  |  |  |  |  |  |
| Lúcuma |  |  |  |  |  |  |  |  |  |  |  |  |  |
|  |  |  |  |  |  |  |  |  |  |  |  |  |  |
|  |  |  |  |  |  |  |  |  |  |  |  |  |  |
|  |  |  |  |  |  |  |  |  |  |  |  |  |  |
| **SEMILLAS** |  |  |  |  |  |  |  |  |  |  |  |  |  |
| Castaña |  |  |  |  |  |  |  |  |  |  |  |  |  |
|  |  |  |  |  |  |  |  |  |  |  |  |  |  |
|  |  |  |  |  |  |  |  |  |  |  |  |  |  |
| **HOJAS** |  |  |  |  |  |  |  |  |  |  |  |  |  |
| Jatata |  |  |  |  |  |  |  |  |  |  |  |  |  |
|  |  |  |  |  |  |  |  |  |  |  |  |  |  |
|  |  |  |  |  |  |  |  |  |  |  |  |  |  |
| **CORTEZAS** |  |  |  |  |  |  |  |  |  |  |  |  |  |
|  |  |  |  |  |  |  |  |  |  |  |  |  |  |
|  |  |  |  |  |  |  |  |  |  |  |  |  |  |
| **OLEO/LÁTEX** |  |  |  |  |  |  |  |  |  |  |  |  |  |
| Miel |  |  |  |  |  |  |  |  |  |  |  |  |  |
| Copaibo |  |  |  |  |  |  |  |  |  |  |  |  |  |
| Goma |  |  |  |  |  |  |  |  |  |  |  |  |  |
|  |  |  |  |  |  |  |  |  |  |  |  |  |  |
| **BEJUCOS** |  |  |  |  |  |  |  |  |  |  |  |  |  |
| Chamairo |  |  |  |  |  |  |  |  |  |  |  |  |  |
| Uña de gato |  |  |  |  |  |  |  |  |  |  |  |  |  |
|  |  |  |  |  |  |  |  |  |  |  |  |  |  |
|  |  |  |  |  |  |  |  |  |  |  |  |  |  |
| **LEÑA** |  |  |  |  |  |  |  |  |  |  |  |  |  |
| Isigo |  |  |  |  |  |  |  |  |  |  |  |  |  |
| Caricari |  |  |  |  |  |  |  |  |  |  |  |  |  |
| Blanquillo |  |  |  |  |  |  |  |  |  |  |  |  |  |
| Pacai |  |  |  |  |  |  |  |  |  |  |  |  |  |
|  |  |  |  |  |  |  |  |  |  |  |  |  |  |
|  |  |  |  |  |  |  |  |  |  |  |  |  |  |
| **MADERA** |  |  |  |  |  |  |  |  |  |  |  |  |  |
| Aliso |  |  |  |  |  |  |  |  |  |  |  |  |  |
| Almendrillo |  |  |  |  |  |  |  |  |  |  |  |  |  |
| Cedro |  |  |  |  |  |  |  |  |  |  |  |  |  |
| Cuta |  |  |  |  |  |  |  |  |  |  |  |  |  |
|  |  |  |  |  |  |  |  |  |  |  |  |  |  |
|  |  |  |  |  |  |  |  |  |  |  |  |  |  |
| **ANIMALES** |  |  |  |  |  |  |  |  |  |  |  |  |  |
| Mono |  |  |  |  | K |  |  |  |  |  |  |  |  |
| Guaso |  |  |  |  | K |  |  |  |  |  |  |  |  |
| Paca |  |  |  |  | K |  |  |  |  |  |  |  |  |
| Jochi |  |  |  |  | K |  |  |  |  |  |  |  |  |
| Chancho |  |  |  |  | K |  |  |  |  |  |  |  |  |
| Taitetú |  |  |  |  | K |  |  |  |  |  |  |  |  |
| Tatu |  |  |  |  | K |  |  |  |  |  |  |  |  |
| Anta |  |  |  |  | K |  |  |  |  |  |  |  |  |
| Pava |  |  |  |  | K |  |  |  |  |  |  |  |  |
|  |  |  |  |  | K |  |  |  |  |  |  |  |  |
|  |  |  |  |  |  |  |  |  |  |  |  |  |  |

1. *Códigos: 1=únicamente/principalmente por la esposa y las mujeres adultas del hogar; 2=tanto mujeres como hombres adultos participan de la misma forma; 3=únicamente/principalmente por el esposo y los hombres adultos del hogar; 4=únicamente/principalmente por las niñas (<15 años); 5=únicamente/principalmente por los niños (<15 años); 6=únicamente/principalmente por los niños (<15 años), niñas y niños participan de la misma forma; 7=todos los miembros del hogar participan de la misma forma; 8= comité comunitario; 9=externos (ejm., empresa, intermediario); 10=ninguna de las anteriores*
2. ¿Realiza alguna actividad extra para aumentar su producción de castaña? Si sí, cuáles de las siguientes actividades realiza?
   1. Limpia las sendas de acceso
   2. Quema debajo de los árboles productores para facilitar la recolección
   3. Transplanta plántulas (natural) y plantines (cultivados)
   4. Limpia alrededor de plántulas y brinzales
   5. Corta lianas o bejucos
   6. Protege las plántulas y brinzales a propósito
   7. Lava las nueces después de cosecharlos
   8. otro

## K. Ingresos derivados del bosque (ingreso de productos forestales procesados)

1. ¿Cuáles son las cantidades y los valores de los productos forestales procesados que los miembros de su hogar produjeron durante **el último año**?

| **1.**  **Prod-ucto**  *(código-producto)* | **2.**  **¿Quién en el hogar realiza el trabajo?^1)^** | **3.**  **Cantidad producida**  **(5+6)** | **4.**  **Unidad** | **5.**  **Uso doméstico (incl. regalos)** | **6.**  **Venta (incl. intercambio o trueque)** | **7.**  **Precio por unidad** | **8.**  **Tipo de mercado**  *(código-mercado)* | **9.**  **Valor bruto (3*7)** | **10.**  **Compra de insumos y pago de mano de obra** | **11.**  **Costos de transporte/comercialización** | **12.**  **Ingreso neto con excepción de los costos de los insumos forestales (9-10-11)** |
| --- | --- | --- | --- | --- | --- | --- | --- | --- | --- | --- | --- |
|  |  |  |  |  |  |  |  |  |  |  |  |
|  |  |  |  |  |  |  |  |  |  |  |  |
|  |  |  |  |  |  |  |  |  |  |  |  |
|  |  |  |  |  |  |  |  |  |  |  |  |
|  |  |  |  |  |  |  |  |  |  |  |  |
|  |  |  |  |  |  |  |  |  |  |  |  |
|  |  |  |  |  |  |  |  |  |  |  |  |
|  |  |  |  |  |  |  |  |  |  |  |  |

1. *Códigos: 1=únicamente/principalmente por la esposa y las mujeres adultas del hogar; 2=tanto mujeres como hombres adultos participan de la misma forma; 3=únicamente/principalmente por el esposo y los hombres adultos del hogar; 4=únicamente/principalmente por las niñas (<15 años); 5=únicamente/principalmente por los niños (<15 años); 6=únicamente/principalmente por los niños (<15 años),niñas y niños participan de la misma forma; 7=todos los miembros del hogar participan de la misma forma; 10=ninguna de las anteriores*

## L. Ingresos ambientales no forestales

1. Además de los productos forestales incluidos en los cuadros anteriores, ¿cuánto de **otros productos silvestres** (ej. De río, pastos naturales, barbechos, etc.) colectó su hogar durante **el último año?**

| **1. Tipo de producto** *(código-producto)* | **¿Dónde fue recolectado?** | | **4. Cantidad colectada (6+7)** | **5. Unidad** | **6. Uso doméstico (incl. regalos)** | **7. Venta (incl. intercambio o trueque)** | **8. Precio por unidad** | **9. Valor bruto (4*8)** | **10. Costos**  **(insumos, pago de mano de obra, comercialización, etc.)** | **11. Ingreso neto (9-10)** |
| --- | --- | --- | --- | --- | --- | --- | --- | --- | --- | --- |
|  | **2. Tipo de sustrato** *(código-tierra)* | **3. Tenencia**  *(código- tenencia)* |  |  |  |  |  |  |  |  |
|  |  |  |  |  |  |  |  |  |  |  |
|  |  |  |  |  |  |  |  |  |  |  |
|  |  |  |  |  |  |  |  |  |  |  |
|  |  |  |  |  |  |  |  |  |  |  |
|  |  |  |  |  |  |  |  |  |  |  |
|  |  |  |  |  |  |  |  |  |  |  |
|  |  |  |  |  |  |  |  |  |  |  |
|  |  |  |  |  |  |  |  |  |  |  |
|  |  |  |  |  |  |  |  |  |  |  |

Nota: Las respuestas en las columnas 2 y 3 deben ser consistentes con las categorías de tierra reportadas.

## LL. Ingreso por salarios

1. ¿Algún miembro del hogar ha recibido salario durante **el último año**?

*Nota: Cada persona puede incluirse en la lista más de una vez para diferentes trabajos.*

| **1. Miembro del hogar (NIP)** | **2. Tipo de trabajo**  *(código-trabajo)* | **3. Días trabajados en el último mes** | **4. Salario por día** | **5. Ingreso total por salarios (3*4)** |
| --- | --- | --- | --- | --- |
|  |  |  |  |  |
|  |  |  |  |  |
|  |  |  |  |  |
|  |  |  |  |  |

## M. Ingresos por negocios propios (ni forestales ni agrícolas)

1. ¿Es usted parte de algún tipo de negocio? De ser así, ¿cuáles han sido los ingresos brutos y los costos relacionados con ese negocio durante **el último año?**

|  | 1. Negocio 1 | 2. Negocio 2 | 3. Negocio 3 |
| --- | --- | --- | --- |
| 1. ¿Cuál es su negocio?^1)^ |  |  |  |
| 1. **Ingreso bruto (ventas)** |  |  |  |
| **Costos:** | | | |
| 1. Compra de insumos |  |  |  |
| 1. Insumos propios además de la fuerza de trabajo (valor equivalente de mercado) |  |  |  |
| 1. Pago de mano de obra |  |  |  |
| 1. Costos de transporte y comercialización |  |  |  |
| 1. Costos de capital (reparación, mantenimiento, etc.) |  |  |  |
| 1. Otros costos |  |  |  |
| 1. **Ingreso neto (2 = ítems 3-8)** |  |  |  |
|  | | | |
| 1. Valor actual de capital |  |  |  |

*Nota: Si el hogar está involucrado en varios negocios, debe llenar una columna para cada negocio*

*1) Códigos: 1=comercio (tienda); 2=procesamiento agrícola;* *3=artesanías; 4=carpintería; 5=motosierrista; 6=otros trabajos especializados; 7=transporte (carro, lancha,…); 8=hotel/restaurante; 9=contratista; 10=prestamista; 19=otro, especificar.*

***N. Ingresos por agricultura – cultivos***

1. ¿Cuáles son las cantidades y los valores de los cultivos que el hogar ha cosechado durante **el** **último año**?

| **1.Cultivos**  *(código-producto)* | **2. Área de producción** (*m^2^)* | **3. Producción total (5+6)** | **4. Unidad (para la producción)** | **5. Uso doméstico (incl. regalos)** | **6. Venta (incl. intercambio o trueque)** | **7. Precio por unidad** | **8. Valor total**  **(3*7)** |
| --- | --- | --- | --- | --- | --- | --- | --- |
| Arroz |  |  |  |  |  |  |  |
| Maíz |  |  |  |  |  |  |  |
| Yuca |  |  |  |  |  |  |  |
| Frejol |  |  |  |  |  |  |  |
| Plátano |  |  |  |  |  |  |  |
| Guineo |  |  |  |  |  |  |  |
| Papaya |  |  |  |  |  |  |  |
| Piña |  |  |  |  |  |  |  |
| Sandia |  |  |  |  |  |  |  |
| Toronja |  |  |  |  |  |  |  |
| Naranja |  |  |  |  |  |  |  |
| Limón |  |  |  |  |  |  |  |
| Lima |  |  |  |  |  |  |  |
| Pacai |  |  |  |  |  |  |  |
| Manga |  |  |  |  |  |  |  |
| Café |  |  |  |  |  |  |  |
| Coca |  |  |  |  |  |  |  |
| Gualusa |  |  |  |  |  |  |  |
| Caña |  |  |  |  |  |  |  |
| Copuazu |  |  |  |  |  |  |  |
| Guayaba |  |  |  |  |  |  |  |
| Camote |  |  |  |  |  |  |  |
| Cayú |  |  |  |  |  |  |  |
| Palta |  |  |  |  |  |  |  |
| Cebolla |  |  |  |  |  |  |  |
| Lechuga |  |  |  |  |  |  |  |
| Tomate |  |  |  |  |  |  |  |
| Perejíl |  |  |  |  |  |  |  |
| Ají |  |  |  |  |  |  |  |
|  |  |  |  |  |  |  |  |
|  |  |  |  |  |  |  |  |

2. ¿Cuáles son las cantidades y valores de los insumos usados en la producción agrícola durante **el último año** (gastos agrícolas en efectivo)? *Nota: Tomar en cuenta todos los cultivos en el cuadro anterior.*

| **Insumos** | **1. Cantidad** | **2. Unidad** | **3. Precio por unidad** | **4. Costo total  (1*3)** |
| --- | --- | --- | --- | --- |
| 1. Semillas |  |  |  |  |
| 1. Fertilizantes |  |  |  |  |
| 1. Pesticidas/herbicidas |  |  |  |  |
| 1. Abono (estiércol) |  |  |  |  |
| 1. Molinos |  |  |  |  |
| 1. Pago de mano de obra |  |  |  |  |
| 1. Maquinaria alquilada |  |  |  |  |
| 1. Transporte/comercialización |  |  |  |  |
| 19. Otros: |  |  |  |  |
| a. Sacos |  |  |  |  |
| b. Machete |  |  |  |  |
| c. Hacha |  |  |  |  |
| d. Lima |  |  |  |  |
| e. Esmeril |  |  |  |  |
| f. Azadón |  |  |  |  |
| g. Pala |  |  |  |  |
| h. Fosa |  |  |  |  |
| i. Máquina para plantar |  |  |  |  |
|  |  | |  |  |
|  |  | |  |  |
| 20. Pago de renta de la tierra |  | |  |  |
|  |  | |  |  |

## O. Ingreso por producción pecuaria

|  | **1. Cantidad hace 1 año** | **2. Vendidos (incl. trueque), vivos o sacrificados** | **3.Sacrificados para uso doméstico (o hacer un regalo)** | **4. Perdidos (robo, muertes,..)** | **5. Comprados o recibidos como regalo** | **6. Nuevo nacido del rebaño** | **7. Cantidad actual (1-2-3-4+5+6)** | **8. Precio por animal adulto** | **9. Valor total final (7*8)** |
| --- | --- | --- | --- | --- | --- | --- | --- | --- | --- |
| 1. **Ganado** |  |  |  |  |  |  |  |  |  |
| Buey |  |  |  |  |  |  |  |  |  |
| Toros |  |  |  |  |  |  |  |  |  |
| Vaca leche |  |  |  |  |  |  |  |  |  |
| Vaca corte |  |  |  |  |  |  |  |  |  |
| 1. Búfalos |  |  |  |  |  |  |  |  |  |
| 1. Cabras |  |  |  |  |  |  |  |  |  |
| 1. Ovejas |  |  |  |  |  |  |  |  |  |
| 1. Cerdos |  |  |  |  |  |  |  |  |  |
| 1. Burros |  |  |  |  |  |  |  |  |  |
| 1. Patos |  |  |  |  |  |  |  |  |  |
| 8. Pollos |  |  |  |  |  |  |  |  |  |
| 1. Caballos/   Yeguas |  |  |  |  |  |  |  |  |  |
| 1. Conejos |  |  |  |  |  |  |  |  |  |
| 10. Otros |  |  |  |  |  |  |  |  |  |

1. ¿Cuántos animales adultos tiene su hogar en la actualidad, y cuántos ha vendido, comprado, sacrificado o perdido durante **el** **último año**?

2. ¿Cuáles son las cantidades y los valores de los servicios y productos de animales que su hogar ha producido durante **el** **último año**?

| **Producto/servicio** | **1. Producción (3+4)** | **2. Unidad** | **3. Uso doméstico (incl. regalos)** | **4. Venta (incl. trueque)** | **5. Precio por unidad** | **6. Valor total**  **(1*5)** |
| --- | --- | --- | --- | --- | --- | --- |
| 1. Carne ^1)^ |  |  |  |  |  |  |
| 1. Leche ^2)^ |  |  |  |  |  |  |
| 1. Mantequilla |  |  |  |  |  |  |
| 1. Queso |  |  |  |  |  |  |
| 1. Manteca |  |  |  |  |  |  |
| 1. Huevos |  |  |  |  |  |  |
| 1. Cuero y pieles |  |  |  |  |  |  |
| 1. Lana |  |  |  |  |  |  |
| 1. Estiércol |  |  |  |  |  |  |
| 1. Energía eólica (molinos) |  |  |  |  |  |  |
| 1. Colmenas de abeja |  |  |  |  |  |  |
| 1. Miel |  |  |  |  |  |  |
| 19. Otro, especificar: |  |  |  |  |  |  |

1. *Asegurarse de que hay correspondencia con el cuadro anterior sobre la venta y el consume de animales*
2. *Solo debe incluirse leche consumida o vendida como tal, no la leche que se usó para queso u otros productos*

3. ¿Cuáles son las cantidades y los valores de los insumos usados en la producción pecuaria durante **el último año** (gastos en efectivo)?

*Nota: Lo importante es obtener costos totales más que las unidades de insumos*

| **Insumos** | **1. Unidades** | **2. Cantidad** | **3. Precio por unidad** | **4. Costos totales (2*3)** |
| --- | --- | --- | --- | --- |
| 1. Forraje |  |  |  |  |
| **Sal** |  |  |  |  |
| **Vitaminas** |  |  |  |  |
| **Otro:** |  |  |  |  |
| 1. Arrendamiento de tierra para pastoreo |  |  |  |  |
| 1. Medicinas, vacunas y otros servicios veterinarios |  |  |  |  |
| 1. Costos de mantenimiento de establos, corrales, etc. |  |  |  |  |
| 1. Pago de mano de obra |  |  |  |  |
| 1. Material proveniente de la granja |  |  |  |  |
| 1. Otro, especificar: |  |  |  |  |

4. Por favor, indicar aproximadamente el porcentaje de forraje consumido por sus animales o traído al terreno por los miembros del hogar.

| **Tipo de tierra para pastoreo o fuente de forraje** | | **3. Porcentaje aproximado** (%) |
| --- | --- | --- |
| **1. Tipo de tierra**  *(código-tierra)* | **2. Tenencia**  *(código-tenencia)* |  |
|  |  |  |
|  |  |  |
|  |  |  |
| Total | | 100% |

| **P. Otras fuentes de ingreso:** 1. Anotar cualquier otra fuente de ingreso que haya recibido en el último año | |
| --- | --- |
| **Tipo de ingreso** | **Cantidad total recibida** |
| 1. Remesas |  |
| 1. Apoyo del gobierno, ONG, organización o similar |  |
| 1. Regalos/apoyo de amigos y parientes |  |
| 1. Pensión |  |
| 1. Pago por servicios ambientales del bosque |  |
| 1. Pago por arrendamiento de la tierra (si es en especie, indicar el equivalente en efectivo) |  |
| 1. Compensación de la empresa maderera o minera (u otro similar) |  |
| 1. Pagos del GUF |  |
| 1. Otro, especificar: |  |

## Q. Evaluación del encuestador/investigador sobre el hogar

*Nota: Esta sección deberá ser completada por el encuestador.*

| 1. Durante la entrevista, ¿el entrevistado sonrió o rió?   *Códigos: 1 = nunca rió ni sonrió (sombrío), 2 = sólo sonrió, 3 = sonrió y rió, 4 = se rió frecuente y abiertamente* |  |
| --- | --- |
| 1. Basado en tus impresiones y en lo que has visto (casa, objetos, etc.), ¿qué tan rico crees que es este hogar comparado con otros hogares de la comunidad?   *Códigos: 1= pobre, 2 = promedio, 3 = rico* |  |
| 1. ¿Cuán confiable es la información **generalmente** brindada por este hogar? *Códigos: 1=pobre; 2=razonablemente confiable; 3=muy confiable* |  |
| 1. ¿Cuán confiable es la información sobre **uso/colecta forestal** brindada por este hogar?   *Códigos: 1=pobre; 2=razonablemente confiable; 3=muy confiable* |  |
| 1. Si la información sobre el bosque no es tan confiable (código 1 arriba), ¿piensas que la información suministrada sobrestima o subestima el uso real del bosque?  *Códigos: 1=subestima; 2=sobrestima; 3= no hay sobre o subestimación sistemática; 4=no sé* |  |
