# Supplementary material for: Socio-ecological costs of Amazon nut and timber production at community household forests in the Bolivian Amazon
Source: PLoS One. 2017 Feb 24;12(2):e0170594. doi: 10.1371/journal.pone.0170594 (PMC5325212; doi:10.1371/journal.pone.0170594)
Supplement: S2 File — Includes Spanish version, the original language in which the agreement was signed upon. (DOCX) [file pone.0170594.s008.docx]

*With Spanish version below…*

**Collaboration agreement for the research “Quest for socio-economic and ecological sustainability of forest management in Bolivian Amazonian communities”**

*Background*: Multiple use forest management constitutes the main activity among Bolivian Amazonian communities. The long postponed recognition of traditional forest use in national policies and of studies compatible with this use did not allow advances at improving community families’ well-being. Due to the potential of the proposed research to contribute to the national policies and to facilitate the implementation of these policies by communities with a community timber management plan (CTMP, a CTMP requires the establishment of permanent research plots to monitor the response of the forest to management interventions), the community has found convenient to support the PhD research project proposed by the For. Eng. Marlene Soriano about the “**Quest for socio-economic and ecological sustainability of forest management in Bolivian Amazonian communities”**. This project seeks to provide reliable information about the state and functioning of community forests upon accounting for the socio-economic characteristics of community households, *campesino* communities and regional communities. This project is to be carried out under a participatory-action research approach.

*Specific objectives of this collaboration agreement:* To establish a participatory monitoring plan of research plots and to obtain reliable information of timber species and Amazon nut population recovery following a range of logging and Amazon nut harvest intensities.

After presenting the PhD research proposal at the *campesino* community of “_______________________”, on date:_________, we agree to collaborate in this research given the following conditions:

*Obligations of the For. Eng. Marlene Soriano,*

The for. Eng. Marlene Soriano guarantees to train selected community members on the fundamental principles of forest tree species growth and on the methods and tools used to carry the proposed research.

Deliver an oral and written report of the activities carried out in the community, together with a copy of the data collected at the end of each fieldwork season.

Consider authorship of the community or of a community representative in Spanish publications resulting of the proposed research.

Deliver two copies of any type of publication made based on the data coming from the community, and to return the results of the proposed research at a workshop addressed to community members.

Deliver a community photo frame reflecting the community’s traditional uses one year after the signing of this collaboration agreement.

*Obligations of the campesino community “__________________”,*

The community guarantees the participation of community members to get trained as long as they receive a just salary that will allow them to bring the daily family income to their home.

The community will provide a camping space for the stay of the researcher and her research team, and will also provide a facility to carry the training of the selected community members.

The community commits to take care and to support the researcher to get further founding for creating a self-financing strategy for the continued monitoring of the permanent research plots.

The community consents the researcher to take photographs of the forest and of the people participating in data collection.

The present collaboration agreement is signed by the president of the community and the researcher in a way to make the commitments and obligations indicated in this document effective.

--------------------------- ----------------------------

Name: For. Eng. Marlene Soriano

Position: Research associate - IBIF

Community: PhD candidate - WUR

**Acuerdo de colaboración en investigación “Búsqueda de la sostenibilidad socioeconómica y ecológica del manejo forestal comunitario en la Amazonía boliviana”**

*Antecedentes*: El uso múltiple del bosque constituye la actividad principal entre las comunidades de la Amazonía boliviana. Debido a la falta de estudios compatibles con la realidad comunitaria en este tema, no se ha podido avanzar en la mejora del bienestar de los hogares comunales ni en el reconocimiento formal de las formas tradicionales de aprovechamiento de sus bosques en políticas nacionales concretas. Por el potencial de la presente investigación para contribuir a la normativa forestal y apoyar en el cumplimiento de los requisitos legales que las comunidades con manejo forestal maderable deben cumplir respecto al monitoreo de la respuesta del bosque al aprovechamiento forestal mediante el establecimiento de parcelas permanentes de investigación, resulta conveniente apoyar el proyecto de investigación de doctorado de la Ing. Marlene Soriano sobre la *”Búsqueda de la sostenibilidad socioeconómica y ecológica del manejo forestal comunitario en la Amazonía Boliviana”*, ya que busca proveer información confiable acerca del estado y funcionamiento de los bosques comunales tomando en cuenta la realidad socio-económica de los hogares, comunitaria y regional; y que además se lo realizará bajo un enfoque de investigación acción-participativa.

*Objetivos específicos del acuerdo de colaboración:* Establecer un plan de monitoreo participativo de parcelas de investigación para obtener información confiable sobre la recuperación del bosque después del aprovechamiento de especies maderables y castaña bajo diferentes intensidades.

Luego de haber sido presentada la propuesta de investigación de doctorado de la Ing. Marlene Soriano, investigadora asociada del Instituto Boliviano de Investigación Forestal-IBIF y estudiante de doctorado de la Universidad de Wageningen-Holanda, en la Comunidad Campesina “_______________________”,en fecha_________, se llega a un acuerdo de colaboración entre partes bajo las siguientes cláusulas:

*De las obligaciones de la Ing. Marlene Soriano,*

La Ing. Marlene Soriano garantiza una capacitación adecuada concerniente a los principios fundamentales del comportamiento de los árboles y métodos de investigación a los miembros que se elijan de la comunidad para apoyar la investigación.

Realizar un reporte oral y presentar un reporte escrito de las actividades realizadas junto a una copia de los datos colectados a la conclusión de cada etapa de campo.

La investigadora deberá considerar la autoría de la comunidad o un miembro representante en publicaciones en español que se realicen en caso la comunidad así lo requiera.

Deberá entregar dos copias de cualquier tipo de publicación realizada en base a los datos colectados, al igual que debe devolver los resultados de la investigación en un taller dirigido a los miembros de la comunidad.

Deberá entregar un retrato comunitario de usos y costumbres al cabo de un año desde la firma del presente acuerdo de colaboración.

*De las obligaciones de la comunidad Campesina “__________________”,*

La comunidad garantizará la participación de las personas elegidas para recibir capacitación, en tanto los participantes reciban un honorario justo que les permita seguir ganando el sustento familiar mientras se capacitan.

La comunidad brindará a la investigadora y a su equipo de investigación un espacio para su camping y para que realice la capacitación a miembros de la comunidad elegidos para capacitarse.

La comunidad se compromete a cuidar y darle seguimiento al monitoreo de las parcelas de investigación instaladas, así como brindar apoyo a la investigadora para conseguir fuentes de financiamiento o crear una estrategia de autofinanciamiento a futuro.

La comunidad autoriza a la investigadora a tomar fotografías en el bosque, y de las personas que se estén capacitando.

Firman el presente acuerdo de colaboración, el presidente de la comunidad y la investigadora a manera de sellar el compromiso y obligaciones estipulados en este documento.

--------------------------- ----------------------------

Nombre: Ing. Marlene Soriano

Cargo: Investigadora Asociada - IBIF

Comunidad: Candidata a Doctorado – WUR

*English version:*
